# Biodegradable and Implantable Triboelectric Nanogenerator Improved by β‐Lactoglobulin Fibrils‐Assisted Flexible PVA Porous Film

**DOI:** 10.1002/advs.202409914

**Published:** 2024-11-11

**Authors:** Yichang Quan, Engui Wang, Han Ouyang, Lingling Xu, Lu Jiang, Lijing Teng, Jiaxuan Li, Lin Luo, Xujie Wu, Zhu Zeng, Zhou Li, Qiang Zheng

**Affiliations:** ^1^ Key Laboratory of Biology and Medical Engineering/Immune Cells and Antibody Engineering Research Center of Guizhou Province, School of Biology and Engineering Guizhou Medical University Guiyang 550025 P. R. China; ^2^ Beijing Institute of Nanoenergy and Nanosystems Chinese Academy of Sciences Beijing 101400 P. R. China; ^3^ School of Nanoscience and Engineering University of Chinese Academy of Sciences Beijing 100049 P. R. China

**Keywords:** biodegradability, physical cross‐linking, PVA aerogel, triboelectric nanogenerator, β‐lactoglobulin fibrils

## Abstract

Triboelectric nanogenerators (TENGs) are highly promising as implantable, degradable energy sources and self‐powered sensors. However, the degradable triboelectric materials are often limited in terms of contact electrification and mechanical properties. Here, a bio‐macromolecule‐assisted toughening strategy for PVA aerogel‐based triboelectric materials is proposed. By introducing β‐lactoglobulin fibrils (BF) into the PVA aerogel network, the material's mechanical properties while preserving its swelling resistance is significantly enhanced. Compared to pure PVA porous film, the BF‐PVA porous film exhibits an eightfold increase in fracture strength (from 1.92 to 15.48 J) and a fourfold increase in flexibility (from 10.956 to 39.36 MPa). Additionally, the electrical output of BF‐PVA in triboelectric performance tests increased nearly fivefold (from 45 to 203 V). Leveraging these enhanced properties, a biodegradable TENG (bi‐TENG) for implantable muscle activity sensing is developed, achieving real‐time monitoring of neuromuscular processes. This innovation holds significant potential for advancing implantable medical devices and promoting new applications in bio‐integrated electronics.

## Graphical abstract

BF‐PVA porous film is prepared by doping BF into PVA to form hydrogel and then freeze‐drying and compression to improve the tensile resistance, bending resistance, swelling resistance, degradation time and electrical properties of PVA. Finally, the degradable triboelectric sensor made of BF‐PVA porous film and Mg film is used to realize the signal analysis of muscle contraction.

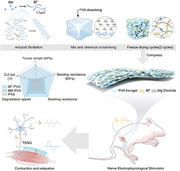

## 1 Introduction

Transient electronics are possible to be fully or partially soluble, degradable and decomposable through electrochemical, mechanical or chemical processes, in a form that allows devices to exist for a controlled period.^[^ ^1^, ^2^, ^3^, ^4^ ^]^ This has led to the emergence of new application areas that balance bio/ecology and electronics, such as biodegradable eco‐devices with little or no waste; medical electronics with no residual problems, which do not require dismantling for surgery.^[^ ^5^, ^6^, ^7^, ^8^, ^9^, ^10^ ^]^ While transient electronic devices show great potential for application, energy devices matching transient characteristics have also become one of the research hotspots in this field, because suitable energy supply is an important guarantee for the normal operation of transient electronic devices. The reported transient energy devices include batteries, capacitors and energy harvesters such as photovoltaics, piezoelectric devices, triboelectric nanogenerators.^[^ ^11^, ^12^, ^13^, ^14^, ^15^, ^16^, ^17^, ^18^ ^]^ Among these, degradable triboelectric nanogenerators (TENGs) show increasing potential due to their unique advantages such as ultra‐low power consumption, ultra‐low cost, wide choice of materials, high processing flexibility and high sensitivity.^[^ ^19^, ^20^, ^21^, ^22^, ^23^, ^24^, ^25^ ^]^

The material is an important part of TENG. The characteristics of the material have a direct impact on the performance and application of TENG, such as surface microstructure, triboelectric properties and mechanical properties.^[^ ^26^ ^]^ Biodegradable triboelectric materials such as chitosan and egg white have good biocompatibility and film‐forming properties, but their mechanical properties are not good. Polyvinyl alcohol (PVA) is one of most commonly used degradable triboelectric material due to its excellent biocompatibility, ease of processing and good mechanical properties.^[^ ^27^, ^28^, ^29^, ^30^ ^]^ However, PVA is highly sensitive to water, and even slight moisture can cause secondary swelling and deformation of the device within a short period, leading to rapid failure of the entire device. This is highly detrimental to its implantable applications.

In order to overcome the problem of water sensitivity of PVA, researchers have used freeze‐thaw method to prepare PVA aerogels. Through freeze‐thaw cycles, the PVA molecular chains become entangled with each other, increasing the crystallinity of the material and thus its resistance to swelling.^[^ ^31^, ^32^ ^]^ However, this method makes the PVA material more brittle and less tough, making it unsuitable as a base material for electronic devices. Therefore, how to obtain degradable PVA triboelectric materials with both swelling resistance and mechanical toughness has become an interesting and important issue, which is valuable for the construction of flexible and degradable implantable TENGs. Aerogels often face the problem of structural fragility, and other research groups have also improved this problem.^[^ ^33^, ^34^ ^]^

Based on this, we propose a toughening strategy by introducing bio‐macromolecules (β‐lactoglobulin fibrils) into the rigid backbone of PVA porous film. The introduction of β‐lactoglobulin fibrils (BF) into can significantly improve the mechanical strength and flexibility of the original PVA gel network by forming macroscopic mechanical interlocking and microscopic chemical bonding with the PVA framework in the formation of the PVA backbone. Compared with pure PVA porous films, BF‐PVA has an 8‐fold increase in fracture strength (1.92 to 15.48 J) and a 4‐fold increase in flexibility (from 10.956 to 39.36 MPa). At the same time, BF‐PVA porous films perfectly retain the original swelling resistance properties and biocompatibility. Moreover, in a relative triboelectric output performance test, the electrical output of BF‐PVA porous films was enhanced by nearly five times (from 45 to 203 V) compared to that of unmodified PVA, which can be attributed to the abundant charged groups additionally provided by BF together with the rich pore structure of the aerogel. Moreover, using the above BF‐PVA porous films we designed a biodegradable TENG (bi‐TENG) as an implantable muscle activity sensor. The real‐time and dynamic monitoring of the neuromuscular regulatory process has been successfully realized, which can be further used in the future to monitor the repair effect after nerve injury.

## 2 Results and Discussion

### 2.1 Overall Flow

**Figure 1:**
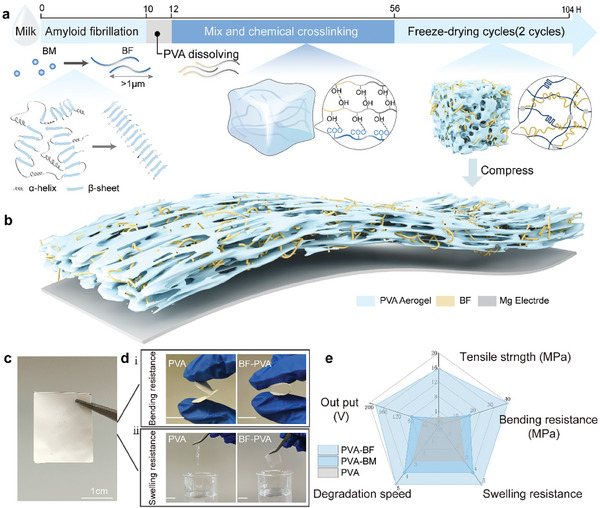
a) Schematic diagrams of preparation process of BF‐PVA aerogel. b) Schematic of BF‐PVA porous films based triboelectric film with Mg electrode. c) Photograph of as fabricated BF‐PVA film. d) Demonstration of the i) fracture and ii) swelling resistance of BF‐PVA film compared with pure PVA film. e) Comparison of the performance of BF‐PVA, BM‐PVA and PVA in terms of tensile resistance, bending resistance, resistance to swelling, degradation rate and output voltage.

As shown in **Figure** **1**a, β‐lactoglobulin is a cost‐effective biomaterial with excellent biocompatibility, which can be efficiently recovered from dairy production waste through relatively straightforward extraction processes. Through unfolding, hydrolysis, and self‐assembly (orderly stacking of β‐sheet structures), the original bovine β‐lactoglobulin monomers (BM) can be transformed into β‐lactoglobulin fibrils (BF), characterized by a unique surface charge, abundant surface functional groups, and good mechanical properties (Figure S1, Supporting Information). By incorporating an appropriate proportion of BF into PVA and conducting multiple freeze‐thaw cycles, the PVA polymer gradually crystallized and chemically bonded with BF through hydrogen bonds, and finally formed a stable hydrogel network structure. Subsequently, the hydrogel material is processed through freeze‐drying to form an aerogel structure. During this process, the PVA matrix and protein fibrils mechanically interlock, further enhancing the mechanical properties of PVA after drying. The BF‐PVA aerogel is compressed into a thin film (172.67±18.23 µm), which, compared to pure PVA films, clearly demonstrates superior fracture and swelling resistance. Through further data measurements, the BF‐PVA porous films exhibit enhanced performance across multiple dimensions (flexibility, bending resistance, swelling resistance, degradation cycle, electrical output), demonstrating its potential for further development into implantable, biodegradable nanogenerators.

### 2.2 Preparation and Characterization of BF‐PVA Flexible Film

**Figure 2:**
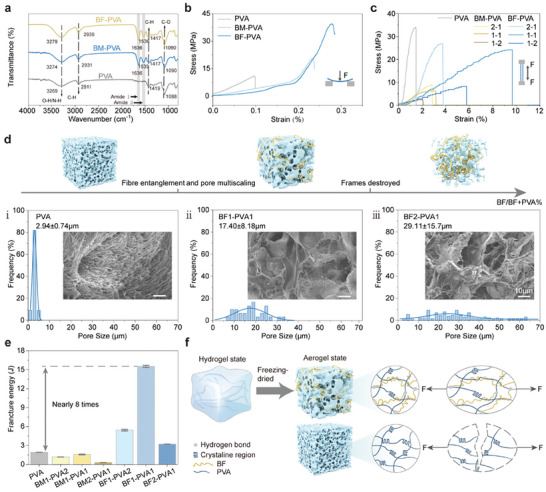
a) ATR‐FTIR spectra showing characteristic peaks for pure PVA, BM‐PVA, and BF‐PVA. b) Stress‐strain curves obtained from three‐point bending tests comparing the mechanical properties of films made from PVA, BM‐PVA, and BF‐PVA. c) Stress–strain curves obtained under tension, showcasing the flexibility and mechanical strength improvements in BF‐PVA compared to PVA and BM‐PVA. d) Schematic representation and SEM images illustrating the microstructure and pore size distribution of aerogels with different BF contents. e) Fracture energy comparison of different porous films samples. f) The potential toughening mechanism of BF‐PVA at both macroscopic and microscopic levels, demonstrating the role of BF in enhancing mechanical properties through mechanical interlocking and chemical bonding with in the PVA matrix.

As shown in Figure S1 (Supporting Information) we first denatured and depolymerized the BM recovered from dairy waste and further self‐assembled it into BF by heating it at PH2, high temperature 80 °C for 10 h. Comparison of the thioflavin T (ThT) fluorescence intensity of the protein solution before and after transformation (Figure S2a, Supporting Information) verified the presence of a large number of β‐sheets, indirectly demonstrating the successful transformation of BM to BF.^[^ ^35^, ^36^ ^]^ Subsequently, we further confirmed the formation of BF by TEM, with fibril lengths of at least 1 µm and diameters of ≈11 nm (Figure S2b, Supporting Information). The dissolved PVA solution was mixed well with BM solution or BF solution in different ratios and poured into the mould (the specific ratios are shown in **Table** **1**). Then after 2 freeze‐thaw cycles to get the hydrogel made by cross‐linking PVA and BM/BF, here we freeze the mixed solution of BM/BF and PVA at −20 °C for 20 h, and then get it to room temperature (24 °C) and leave it for 4 h, which is called a freeze‐thaw cycle. And then freeze‐dried to obtain an interoperable porous aerogel consisting of a PVA framework interwoven with protein fibrils (Figure 1a). The ATR‐FTIR spectra showed that the PVA sample doped with BM/BF would show the characteristic peaks of amide I and amide II (1636 – 1539 cm^−1^), which were significantly shifted to higher wavelengths with respect to the characteristic peaks of amide I and amide II of pure BM/BF (1628 – 1508 cm^−1^) (**Figure** **2**a), which indicated that PVA and BF/BM formed by hydrogen bonding through the stabilized system.

**Table 1:** Ratio of PVA‐BF/BM.

Description of Sample [5.14 wt%]	PVA [12 wt%]	BLGM [4 wt%]	BLGF [4 wt%]	Deionized Water
PVA	3.00 ml	0.00 ml	0.00 ml	4.00 ml
PVA2‐BF1	2.00 ml	0.00 ml	3.00 ml	2.00 ml
PVA1‐BF1	1.50 ml	0.00 ml	4.50 ml	1.00 ml
PVA1‐BF2	1.00 ml	0.00 ml	6.00 ml	0.00 ml
PVA2‐BM1	2.00 ml	3.00 ml	0.00 ml	2.00 ml
PVA1‐BM1	1.50 ml	4.50 ml	0.00 ml	1.00 ml
PVA1‐BM2	1.00 ml	6.00 ml	0.00 ml	0.00 ml

Cyclic freezing and thawing is a commonly used method to make physical cross‐linking of PVA. This method induces the creation of many crystalline regions between the PVA molecular chains, which significantly increases the stiffness and resistance to swelling of the material; on the other hand, this also reduces the flexibility of the material and makes it brittle. In this study, we formed amyloid fibrils (BF) with very good biocompatibility and mechanical properties by self‐assembly up to 10 µm in length (as shown in Figure S2b, Supporting Information taken by TEM). BF doped into the PVA matrix plays a role like that of reinforcing fibrils in composites. When the film is subjected to bending or stretching, these fibrils can withstand some of the stresses, dispersing the stress concentration and preventing the expansion of cracks, thus improving the overall toughness of the material (Figure 2f). We compared the stress‐strain curves of three different types of PVA porous films, including pure PVA(PVA), BM doped PVA (BM‐PVA), and BF doped PVA (BF‐PVA), by a three‐point bending test. As shown in Figure 2b, compared to the brittle and rigid characteristics exhibited by PVA, the BF‐PVA demonstrates superior flexibility and a greater resistance to fracture caused by bending. The modulus of elasticity for pure PVA after freeze‐thawing was measured at 1.2 MPa. By XRD mapping analysis, all samples had a characteristic peak at 19.8°, which indicated the presence of PVA crystals in all of them (Figure S14, Supporting Information). And BF‐PVA and BM‐PVA have a significantly lower peak intensity at 19.8° due to the addition of proteins (BM / BF), which indicates a decrease in the degree of crystallinity, resulting in a great restoration of the flexibility of the films. However, the incorporation of protein (BM/BF) reduced the crystalline regions and increased hydrogen bonding, resulting in a significant improvement in the film's flexibility. Specifically, the modulus of elasticity for BF‐ PVA decreased to 0.3 MPa. Additionally, when comparing the folding behavior of the two films, the pure PVA film broke easily, while the BF‐PVA film remained intact and fully unfolded after bending (Figure 1di, Video S1, Supporting Information).

Macroscopically, PVA aerogels have obvious wrinkles and deformation, while BF‐PVA surfaces are smoother and have no wrinkles (Figure S4, Supporting Information). Then we also observed the microscopic pore structure of the aerogel cross‐section by SEM and counted the pore size distribution of BF‐PVA with different doping ratios (Figure S15, Supporting Information). In particular, the porosity gradually increased with increasing BLGF content (from 62.06 ± 7.02% to 89.71 ± 0.8%) and the pore sizes ranged from relatively homogeneous sizes (2.94 ± 0.74 µm) (Figure 2di) to a multi‐sized pore size distribution (17.4 ± 8.18 µm) (Figure 2diii). This may be since the interaction between BF and PVA (hydrogen bond) hinders the rearrangement and crystallization of PVA molecular chains during the gel formation process. This change in pore structure reduces the formation of crystalline zones but increases the deformable space of the material, allowing for better absorption and dispersion of energy when a force is applied, resulting in improved toughness. For the enhancement of the toughness of the material, we made a data‐based comparison using Mark‐10 for different doping ratios and the enhancement of the pull‐up performance of BF relative to BM (Figure 2c). As indicated by the tensile strain–stress curve, the introduction of BF greatly improved the mechanical properties of the material. However, with the further increase of BF content, the mechanical properties tended to decrease again, which could be attributed to the fact that the addition of too much BF damaged the basic PVA framework (Figure 2d), and this structural damage affects the overall integrity of the material, making it more prone to structural instability and rupture when subjected to stress. For example, the tensile strength of the material was 24.19 MPa when the BF content was 50%, whereas the tensile strength decreased to 8.23 MPa when the BF content was increased to 66.67%. Therefore, we need to choose the proper BF doping ratio to ensure the flexibility of the films. After counting the fracture energies of the materials, we found that the best mechanical properties were obtained at BF1‐PVA1, and there was nearly 8 times enhancement relative to freeze‐dried pure PVA (Figure 2e).

Considering the subsequent sensing application scenarios of this material to face clinical physio‐mechanical signals, we calculated the Young's modulus (0.3 MPa) at BF1‐PVA1 based on Figure 2c, which is similar to the softness of cardiovascular muscles and other organs in the human body, as compared to the human organs (Figure S6, Supporting Information). Moreover, we concerned the hydrophilicity of the samples by water contact angle test (Figure S7, Supporting Information), and the samples with BF/BM added had better hydrophilicity compared to the PVA samples.

### 2.3 Electrical Performance of BF‐PVA as a Triboelectric Material

**Figure 3:**
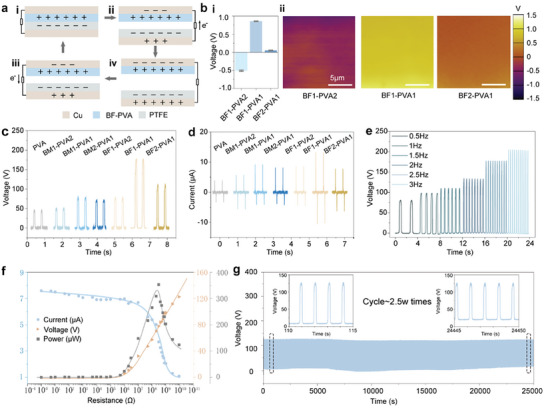
a) Schematic diagram of the relative triboelectric output performance test system based on the classical contact‐separation principle. b) Surface potentials and statistical plots of films with different doping ratios. Different sample films with PTFE contact‐separation generated by c) voltage output, d) current output. BF1‐ PVA1 with PTFE contact‐separation resulting in e) frequency response, f) peak voltage, peak current, and power at different impedances, g) 2.5 W cycles of stability.

We conducted a detailed investigation into the electrical properties of BF‐PVA as a triboelectric material. A test system based on the classical contact‐separation mode of TENGs was utilized to compare the triboelectric properties of various porous films samples (PVA, BM‐PVA, BF‐PVA), and to identify the optimal sample. As illustrated in **Figure** **3**a, when polytetrafluoroethylene (PTFE) comes into perpendicular contact with the sample, electrons transfer from the sample to the PTFE due to the difference in their triboelectric properties, specifically their ability to attract charge. And then, as the separation distance between the positively charged sample and the negatively charged PTFE increases, electrons flow through an external circuit between the two triboelectric layers until the potentials reach equilibrium.

In this test, the open‐circuit voltage of the BF‐PVA was boosted by a factor of nearly four (from 45.7 to 177.65 V) relative to the pure PVA freeze‐thawed sample (Figure 3c). We analyze the reasons for this. From a structural point of view, the inhomogeneity of the pore structure of PVA aerogels, increases the roughness of the material surface and the actual contact area. Atomic force microscopy (AFM) observations of their surface roughness reached the same conclusion (Figure S16a, Supporting Information). The average roughness of BF‐PVA was higher than that of BM‐PVA and PVA (Figure S16b, Supporting Information). And during friction, a larger contact area helps in more charge transfer, thus increasing the output voltage.^[^ ^37^ ^]^ We also confirm this conclusion by comparing the output of porous film and smooth film (Figure S23, Supporting Information). On the other hand, the entanglement and network structure of fibrous material in aerogels may provide more paths for transporting charges due to friction. These fibrils enhance the charge collection and transfer efficiency, thus increasing the output voltage. Thinking in terms of surface chemical properties, the introduction of BF provides more binding sites for electrons compared to the more hydrogen bonds exposed by BM.^[^ ^38^ ^]^ The stable structure of tyrosine (Tyr) and tryptophan (Trp) in the protein backbone, formed by Π‐Π stacking, also provides an excellent bridge for the flow of electrons.^[^ ^39^ ^]^ And the electropositive is highest at BF1‐PVA1, which is consistent with macroscopic electrical output phenomena. We also explored the dielectric constant and dielectric loss of PVA before and after modification. The dielectric constant of PVA, BM‐PVA and BF‐PVA samples gradually increased, and the dielectric loss after modification was significantly lower than that before modification. In TENG, the increase of dielectric constant is of great help to the output characteristics, which also proves that BF modification can improve the output performance of PVA (Figure S17, Supporting Information). The above conjecture is corroborated by our observation through KPFM that its surface electropositivity increases with increasing BF content (Figure 3b). Based on the above three reasons, the triboelectric properties of PVA samples were significantly improved by BF (Figure 3d; Figure S8, Supporting Information).

After determining that the mechanically and electrically optimal ratio is BF1‐PVA1, we also observed the corresponding frequency response of the device at 0.5 to 3 Hz (Figure 3e; Figure S9, Supporting Information). For the relationship between output and film thickness/input pressure/distance we also made an exploration (Figures S19–S21 and Table S1, Supporting Information).When the device is subjected to an applied load, the voltage increases and the current decreases as the load resistance increases. The power under the corresponding load resistance is obtained from the test data and reaches a maximum power of 352.9 µW at ≈300 MΩ (Figure 3f). Finally, we evaluated the excellent stability of the device. We recorded the output of BF‐PVA for 21 days (Figure S22, Supporting Information), after ≈25 000 contact separation cycles at 1 Hz (Figure 3g).

### 2.4 Fabrication of bi‐TENG and Its Application as Self‐Powered Wearable/Implantable Sensors

**Figure 4:**
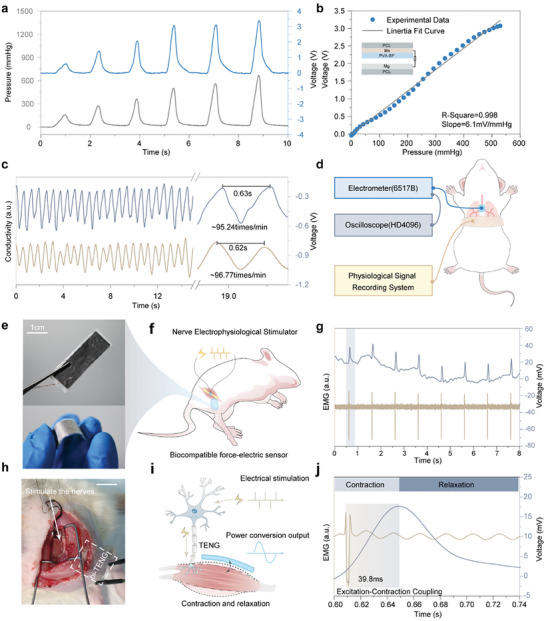
Output voltage of bi‐TENG driven by pneumatic pressure compared to pressure‐driven a) signal and b) linearity. c) Comparison of the respiratory signals received by bi‐TENG's commercial sensors and signal amplification plots. d) Schematic diagram of the respiratory test device. Muscle sensing test of e) bi‐TENG photographs and f) experimental schematic h) implant photographs. g) EMG signal to sensor signal correspondence and j) a cycle of signal amplification plots. i) Schematic diagram of muscle contraction and relaxation by electrical stimulation through nerve conduction.

After identifying the optimal doping ratio of BF for improving mechanical and electrical properties, the BF‐PVA film was utilized as triboelectric layer and sputtered a layer of Mo on its backside as electrode layer. Then, it was assembled with Mg film, which served as both triboelectric layer and electrode layer, to form bi‐TENG. Finally, the entire device is encapsulated with polycaprolactone (PCL) to facilitate its use in implantable studies (**Figure** **4**b). The fabricated bi‐TENG can be used as a self‐powered wearable or implantable sensor. Before formal application, we first tested its sensing performance by comparing it with a commercial stress sensor. As shown in Figure **4**a,b, the bi‐TENG sensor has good sensitivity (6.1 mV mm Hg^−1^) and excellent linearity (R^2^ = 0.998) at driving pressures in the range of 0–550 mm Hg^−1^, which is crucial for the subsequent wearable and implantable sensing applications.

For in vitro physiological activity monitoring of sensors, we focus on respiration, which is very important in human activity. The bi‐TENG and commercial mechanical sensors were fixed at the thorax, and following the rat's breathing, the rise and fall of the thorax drove the signals from the sensors in real time (Figure 4d). Specifically, during inspiration, the thorax squeezes the bi‐TENG, causing the voltage signal to rise (nearly 400 mV), and next, during expiration, the contraction of the thorax causes the bi‐TENG to be released and the voltage signal to decrease. The dynamic respiratory signals obtained by bi‐TENG showed a high degree of waveform and frequency agreement with those obtained by commercial sensors (frequency error of only 1.58%) (Figure 4c). Thus, bi‐TENG has the potential to be used for self‐driven wearable sensing.

For in vivo physiological activity monitoring of sensors, we consider the monitoring of muscle movements. For the assessment of injured nerve repair, the electrodes of an EMG recording device usually need to be lodged into the muscle, this test method does not allow for long term, easy recording.^[^ ^40^ ^]^ We implanted bi‐TENG subcutaneously in the leg to sense the tension and contraction of the muscle due to neural control (Figure 4e–j). Specifically shown in Figure 4j, when electrical stimulation is applied to a nerve, the muscle undergoes diastole and squeezes the bi‐TENG, which is mapped to an increase in voltage signal. And when the muscle contracts back to its initial state, bi‐TENG is released, mapping to a decrease in the voltage signal. And we can time the time between the generation of an action potential and the onset of muscle contraction. This process is how muscle fibrils, after receiving an electrical signal from the nervous system, convert this signal into an actual mechanical contraction action, representing the process of Excitation‐Contraction Coupling. Thus, bi‐TENG can be used to assess the later stages of nerve repair for the assessment of muscle motor control (Figure S10, Supporting Information).

### 2.5 Biosafety Assessment In Vitro and In Vivo

**Figure 5:**
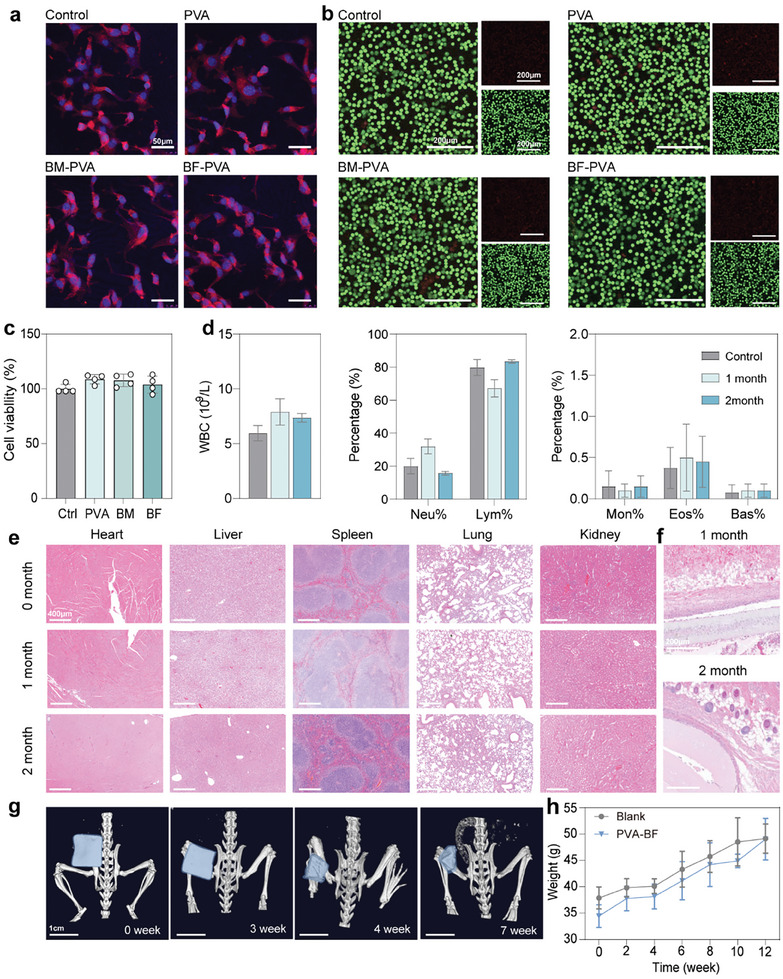
a) Cell morphology of L929 cells grown on the surface of different materials for 3 days. b) Live and dead fluorescence staining of L929 cells grown on different materials for 24 h. c) Comparison of blood routine between non‐implanted mice and mice implanted with BF‐PVA at 1 month and 2 months after implantation. d) The percentage of surface cell viability of different materials relative to the control group. Mice implanted with BF‐PVA samples at 1 month and 2 months after implantation e) heart, liver, spleen, lung and kidney sections and f) skin sections at the implanted site. g) Morphological changes of implanted BF‐PVA in mice for 7 weeks. h) Changes in weight of mice implanted with BF‐PVA and the control group at 12 weeks.

Biocompatibility testing of implantable devices is essential. The biosafety of the materials was determined by observing the morphology, value added, and viability of the cells on the surface of PVA, BM‐PVA, and BF‐PVA in comparison with a blank control group. Observation of cell morphology after 3 days showed very good adhesion and value addition on BF‐PVA (**Figure** **5**a; Figure S11, Supporting Information). This may be due to the fact that BF contributes to cell adhesion and migration. After 24 h of incubation on the surface of the different materials, the cells were subjected to live‐dead staining and counted, which showed that almost all of them consisted of green live cells, with very few red dead cells, and there was no significant difference between the groups (Figure 5b,c) The BF‐PVA films were cut into 1×1cm^2^ sizes and implanted subcutaneously on the back of mice. The status of the film in the mice and its biocompatibility for the mice were continuously recorded for 2 months after implantation. The heart, liver, spleen, lung, kidney and skin around the implant site were histologically stained at 1 month and 2 months after implantation (Figure 5e,f). Blood routine examination was performed by testing the total number of white blood cells (WBC) and the percentage of five white blood cells, neutrophils (Neu), lymphocytes (Lym), monocytes (Mon), eosinophils (Eos), and basophils (Bas) (Figure 5d). The comprehensive results showed no obvious inflammation. And the weight of implanted and non‐implanted mice increased steadily (Figure 5h). The film remained in good shape in vivo for almost a month in mice as observed by CT, and its state as observed by skin dissection was consistent with its resistance to swelling in water in vitro (Figure 5g; Figures S4b,S18, Supporting Information).

## 3 Conclusion

In summary, we developed bi‐TENG using a bio‐macromolecule‐enhanced PVA porous films (BF‐PVA) to address the challenges of swelling resistance and mechanical brittleness in PVA‐based TENGs. By introducing β‐lactoglobulin fibrils into the PVA aerogel network, we achieved a significant enhancement in the material's mechanical properties, with an eightfold increase in fracture strength and a fourfold increase in flexibility compared to pure PVA porous films. Additionally, the BF‐PVA retained its swelling resistance and biocompatibility, making it suitable for implantable applications. The electrical output of the BF‐PVA was nearly five times higher than that of unmodified PVA, demonstrating its potential as an efficient energy source for transient electronics. The bi‐TENG was successfully employed as an implantable muscle activity sensor, enabling real‐time monitoring of neuromuscular processes, which is crucial for applications such as nerve injury repair monitoring.

The BF‐PVA's performance is supported by its advantageous properties, including: i) enhanced mechanical strength and flexibility; ii) robust swelling resistance; iii) high electrical output, and iv) biocompatibility. The bio‐derived β‐lactoglobulin fibrils contribute to the material's cost‐effectiveness, easy availability, and safety, further enhancing its suitability for biomedical applications. Given these results, we believe the BF‐PVA based bi‐TENG will not only advance the development of biodegradable and implantable electronics but also open new avenues in bio‐integrated and eco‐friendly technologies, contributing to the growing field of sustainable and transient electronics.

## 4 Experimental Section

### Materials

Poly(vinyl alcohol) (PVA, CAS: 9002‐89‐5, 1799), β‐lactoglobulin (β‐BLG, CAS:9045‐23‐2, 95%) and PCL powder (Mw 6500) was purchased from Aladdin. The SD rats were purchased from Beijing Vital River Laboratory Animal Technology Co. Ltd.

### Preparation of β‐Lactoglobulin Fibrils (BF)

BLGMs were dissolved in Milli‐Q water to produce a solution of 4 wt%. The pH was then adjusted to 2.0 by adding HCl solution. The resulting BM solution was transferred to a sealed glass bottle, heated at 80 °C for 10 h with magnetic stirring, and subsequently cooled in an ice‐water bath and stored at 4 °C. This solution was designated BF.

### Preparation of BF‐PVA Films

The PVA/BF films were fabricated by a repeated freeze‐thawing process and a lyophilization and compression process. PVA solution (12 wt.%) was prepared and stored at room temperature. The prepared BF solution (4 wt.% total concentration) or BLGM solution (4 wt.%) was mixed with PVA solution (12 wt.%) under vigorous stirring to obtain a well‐mixed solution. The total polymer concentration of the mixed solution was 6 wt.%. Table 1 shows the detailed formulations and corresponding names of each sample. After standing for a period of time to remove air bubbles, the resulting solution was poured into petri dishes of the same size (diameter = 3.5 cm). The solution was subjected to two consecutive freeze‐thaw cycles (freezing at −20 °C for 21 h and thawing at room temperature for 3 h), then was lyophilized and pressed into a flexible film.

### The Fabrication of TENG

The prepared films were cut into 1.5 cm x 2.5 cm rectangles and then a layer of Mo electrode was sputtered on one side of the composite film by plasma enhanced chemical vapor deposition (Si 500D, SENTECH, Germany) for 1 h. Lead a copper wire from the Mo electrode side. Then cut the thin Mg film to the same size as both another triboelectric film end electrode, and lead it with copper wire. The two layers are then glued together with a 2 mm spacer. The PCL film is used as an encapsulation layer for the sensor by hot pressing. PCL film was used as an encapsulation layer for the sensor using hot pressing. After dissolving PCL powder in methylene chloride solution to make a 20% solution, the PCL film was made by using a homogenizer in a fume hood.

### In Vitro Electrical Test of TENG

In order to test the electrical properties of the materials with different doping ratios, a linear motor was used to allow the test films to be periodically contact‐ separated from the PTFE film. The triboelectric open‐circuit voltage (V_OC_), short‐circuit current (I_SC_) and transferred charge (Q_SC_) were tested by an electrometer (Keithley 6517B) and recorded by an oscilloscope (Teledyne LeCroy HD 4096).

### Characterization Measurements

To study the denaturation from BM to BF, amyloid fibrillar protein morphology was observed by atomic force microscopy (AFM) and transmission electron microscopy (TEM). The functional groups of the samples were tested by Fourier transform infrared spectroscopy (FTIR), and the secondary structure of the samples was characterized by circular dichroism (CD). The microscopic pore structure of the surface and cross‐section of the BM‐PVA and BF‐PVA membranes were characterized by scanning electron microscopy (SEM SU8020), and the pore size distribution was statistically analyzed by Image J software. Tensile properties of composite films in various ratios were tested by Mark10. Contact angle measurements were performed on samples at room temperature using a Contact angle meter (XG‐CAM). 10 µl of Milli‐Q water droplets were added to the sample surface, the image was saved, and the contact angle was analyzed.

### Biocompatibility Evaluation of TENG

L929 cells were inoculated in 24‐well cell culture plates and cultured in standard Dulbecco's modified Eagle's medium (DMEM, Gibco) with 1% penicillin/streptomycin (Gibco) and 10% fetal bovine serum (FBS, Gibco) for 48 h at 37 °C and 5% CO_2_ atmosphere, and the DMEM medium obtained served as a cell biocompatibility blank control group for compatibility test. In the experimental group, the suspension was obtained by soaking the sterilized samples in DMEM for 24 h, and then the cells were seeded in the suspension and then subjected to live‐dead staining (37 °C, 20 min), and then the fluorescence images of the cells were observed using a fluorescence microscope (DM 6000). Moreover, in order to quantify the cell viability level of different experimental groups, L929 cells were washed three times with 1xPBS and then incubated with medium containing 10% CCK‐8 reagent for 2 h, and finally the cell activity was determined by testing the absorbance using a microplate absorbance assay instrument.

For the growth morphology of cells on different substrates, we stained their cytoskeleton and nucleus to observe. After L929 cells were cultured on different samples for 3 days, they were immersed in 4% paraformaldehyde solution for 10 min to fix. Finally, they were stained with Rhodamine‐Phalloidin (Abcam) and DAPI (4′, 6‐diamidino‐2‐phenylindole, Solarbio) staining solution at 37 °C. After washing with PBS for 3 times, the cells on the slides were observed using a laser confocal microscope (TCS SP8, Leica).

For the tissue compatibility of the material in vivo, we implanted the BF‐PVA membrane subcutaneously into SD mice. The skin of the implanted site on the back of the mice and organs was taken for HE staining at 1 and 2 months after implantation. At the same time, the condition of the subcutaneous material on the back of the mice was photographed. The anti‐swelling and degradation of the materials in vivo were observed and recorded by micro‐CT. At the same time, the degradation and swelling of the material in phosphate buffer saline (1xPBS) at 37 °C for 3 months were recorded. The tail venous blood of mice to analyze the level of inflammatory cells in the blood was collected. The blood routine examination was performed using the animal blood cell analyzer (52VET, Beijing Keyue Huacheng).

### TENG Senses Force Signals In Vivo and In Vitro of Animals

All animal experiments were carried out in accordance with the requirements of the Beijing Institute of Nanoenergy and Systems (2023016LZ) under the national standards of Laboratory Animal Requirements of Environment and Housing Facilities (GB14925‐2001).

The respiratory signal monitoring experiment was carried out by fixing the commercial respiratory sensor and TENG at the thoracic part of the rat with a strap, and recording the signals with a physiological signal recording system (MP150) and an electrometer (Keithley 6517B), respectively.

For the muscle motion detection experiment, the sterilized TENG was implanted in the middle of the leg muscle and subcutaneous tissue of anesthetized rats to closely perceive the changes of muscle contraction and relaxation, and the electrical signal was tested by an electrostatic meter and recorded by an oscilloscope. The muscle movement applied additional electrical stimulation to the sciatic nerve of the rat through the cuff electrode, and the electrophysiological recorder (Biopac 347 MP150) was used to record the EMG signal of the muscle induced by the nerve conduction stimulation. Specifically, the positive and negative electrodes were inserted under the leg muscles of the rats, and then the grounding electrode was inserted far away from the implantation.

## Conflict of Interest

The authors declare no conflict of interest.

## Author Contributions

Y.C.Q., E.G.W., and O.H. contributed equally to this work. H.O., Z.Z., Z.L., and Q. Z. conceptualized and adapted the topics and experiments. Y.C.Q. and, E.G.,W. made samples and carried out animal experiments. L.J.T. supported the guidance of material characterization. L.L.X. and J.X.L. completed the cell experiments. J.L., X.J.W., and L.L. process the data and draw a schematic diagram. Y.C.Q., E.G.W., and O.H. completed the paper writing and was guided by H.O., Z.Z., Z.L., and Q.Z.

## Supporting information



Supporting Information

Supplemental Video 1

## Data Availability

The data that support the findings of this study are available from the corresponding author upon reasonable request.
