# Supplementary material for: Biodegradable and Implantable Triboelectric Nanogenerator Improved by β‐Lactoglobulin Fibrils‐Assisted Flexible PVA Porous Film
Source: Adv Sci (Weinh). 2024 Nov 11;12(24):2409914. doi: 10.1002/advs.202409914 (PMC12199574; doi:10.1002/advs.202409914)
Supplement: Supplementary file 1 — Supporting Information [file ADVS-12-2409914-s002.docx]

Supporting Information

**Biodegradable and Implantable Triboelectric Nanogenerator Improved by β-Lactoglobulin Fibrils-assisted Flexible PVA porous films**

*Yichang Quan#, Engui Wang#, Han Ouyang#*, Lingling Xu, Lu Jiang, Lijing Teng, Tianbao Qian, Jiaxuan Li, Lin Luo, Xujie Wu, Zhu Zeng*, Zhou Li*and Qiang Zheng*.*


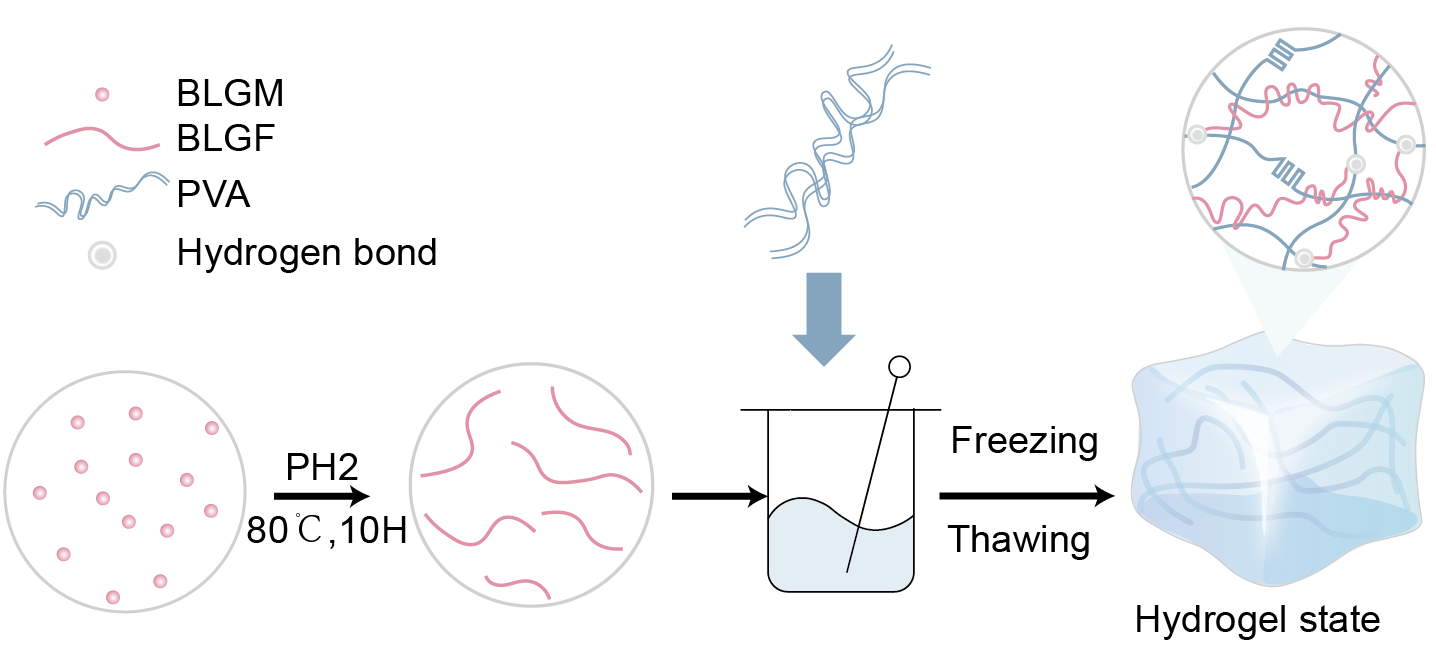


**Figure S1. Preparation of PVA-BF hydrogel.** BM is heated at 80 °C for 10 hours under the condition of pH = 2, and it will expand, hydrolyze and self-assemble to form BF. The BF solution is mixed with PVA in different proportions, and the hydrogel is formed by repeated freeze-thaw cycles after sufficient stirring.


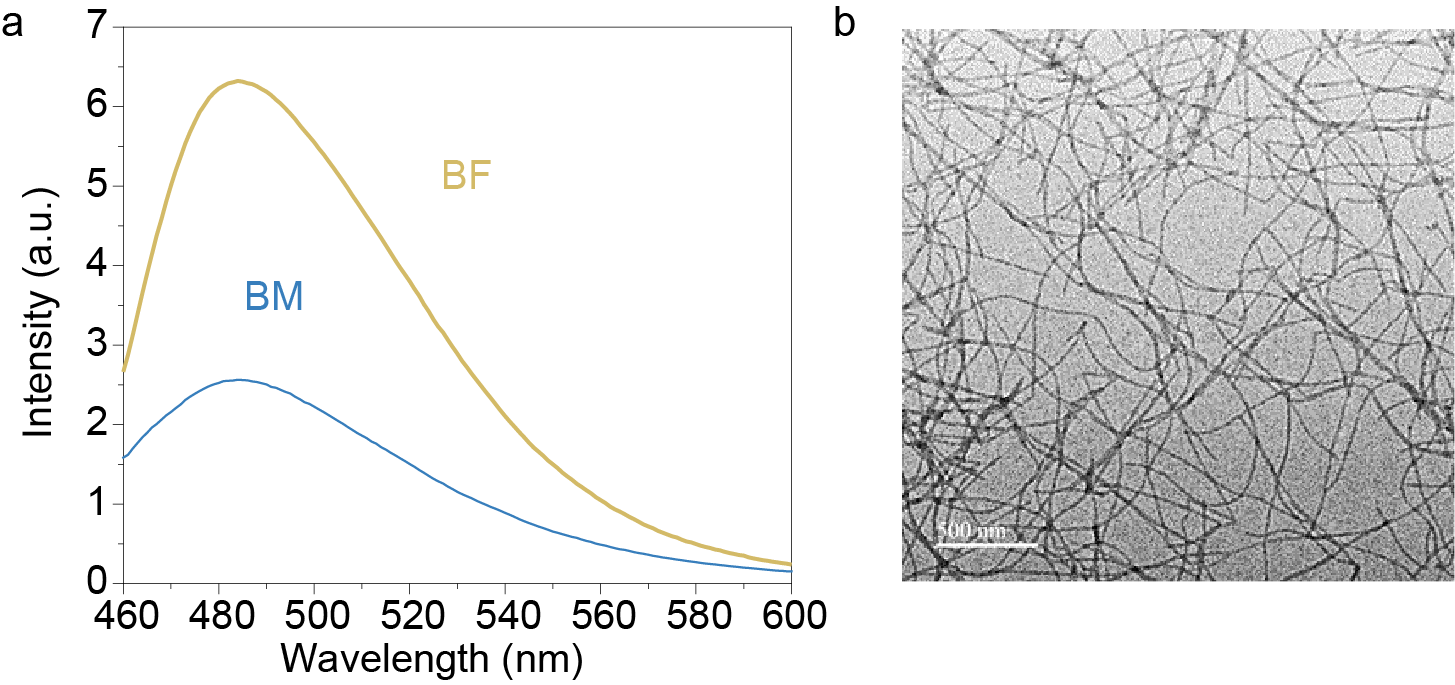


**Figure S2.** **Characterization of BF. a)** Comparison of ThT fluorescence intensity between BM and BF. ThT is a fluorophore that can increase the quantum yield after binding to the β-sheet structure. The fluorescence intensity of BF is much higher than that of BM, indicating that BM is transformed into BF through the accumulation of β-sheet. **b)** TEM images of BF samples.

**
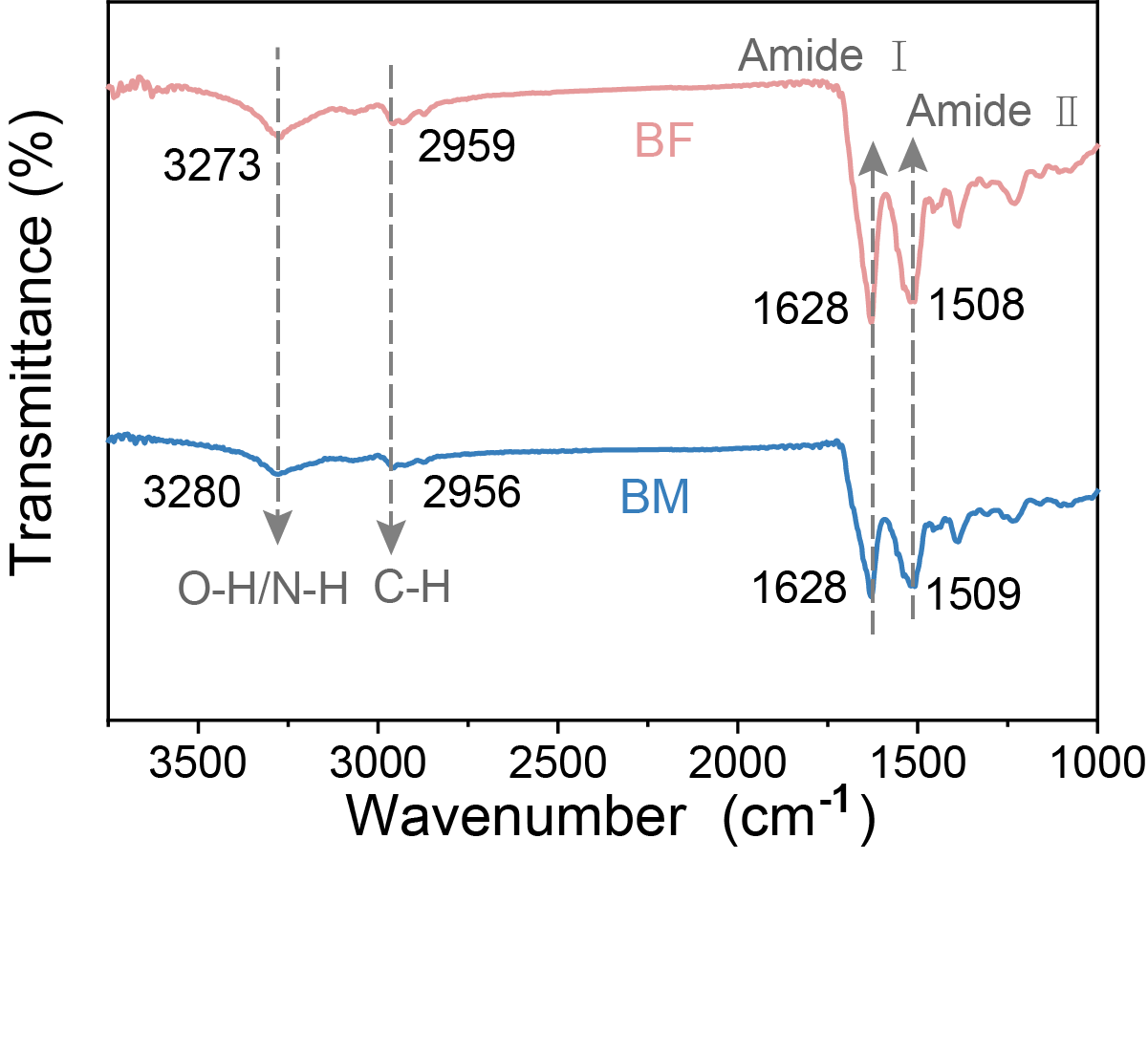
**

**Figure** **S3. ATR-FTIR spectra of pure BM and BF.** The functional groups in BM and BF were detected by ATR-FTIR spectroscopy, and the positions of characteristic peaks in BM and BF curves were similar.


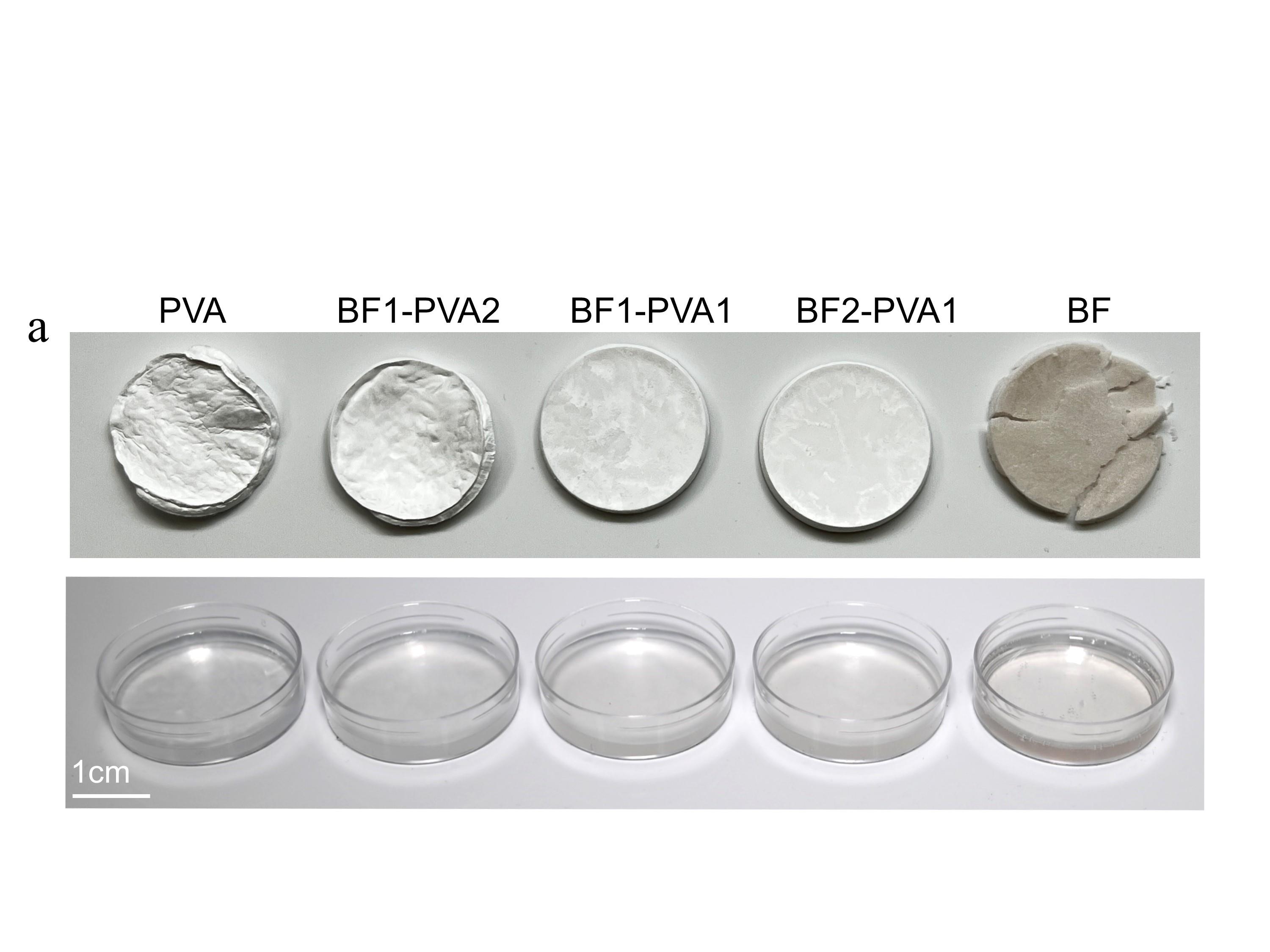

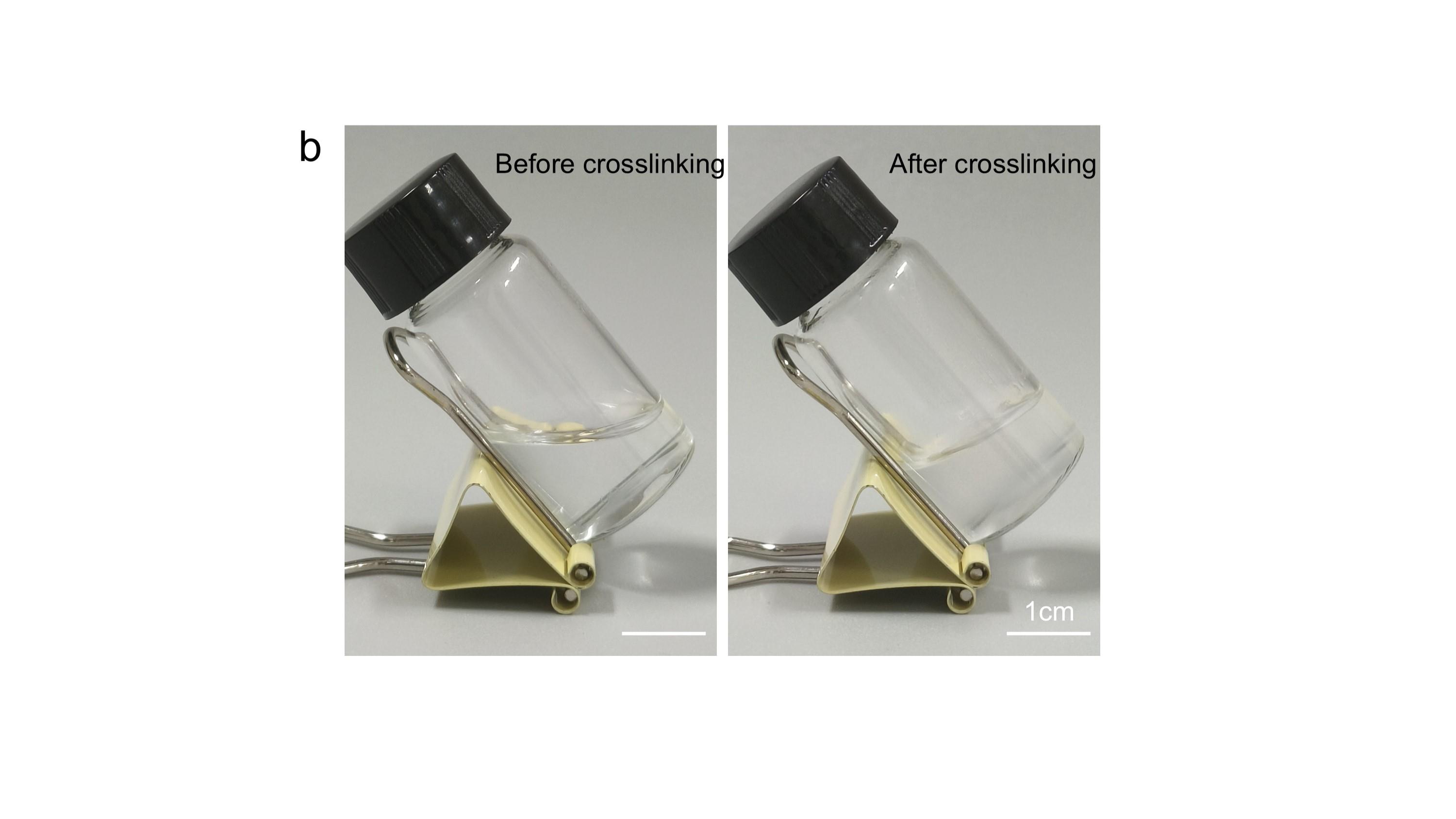


**Figure** **S4. a)** Comparison of hydrogel state and aerogel state of PVA and BF mixed in different ratios. The surface of pure PVA is more wrinkled than the mixed surface of PVA and BF. The lower the proportion of BF, the more wrinkled the surface. b) The mixed liquid before freeze-thaw crosslinking is in a flowing state, but after freezing crosslinking and thawing at room temperature, a stable hydrogel state is formed.





**Figure** **S5. SEM images and corresponding pore size distributions of aerogel sections formed by mixing PVA and BF in different proportions.** From the SEM images, it can be seen that the larger the percentage of BF, the more sparse the pores are. The pore size distribution map exhibits that with the increase of BF content, the pore size distribution is gradually dispersed, and the pores go from a single small scale to a multiscale with uneven sizes.

**
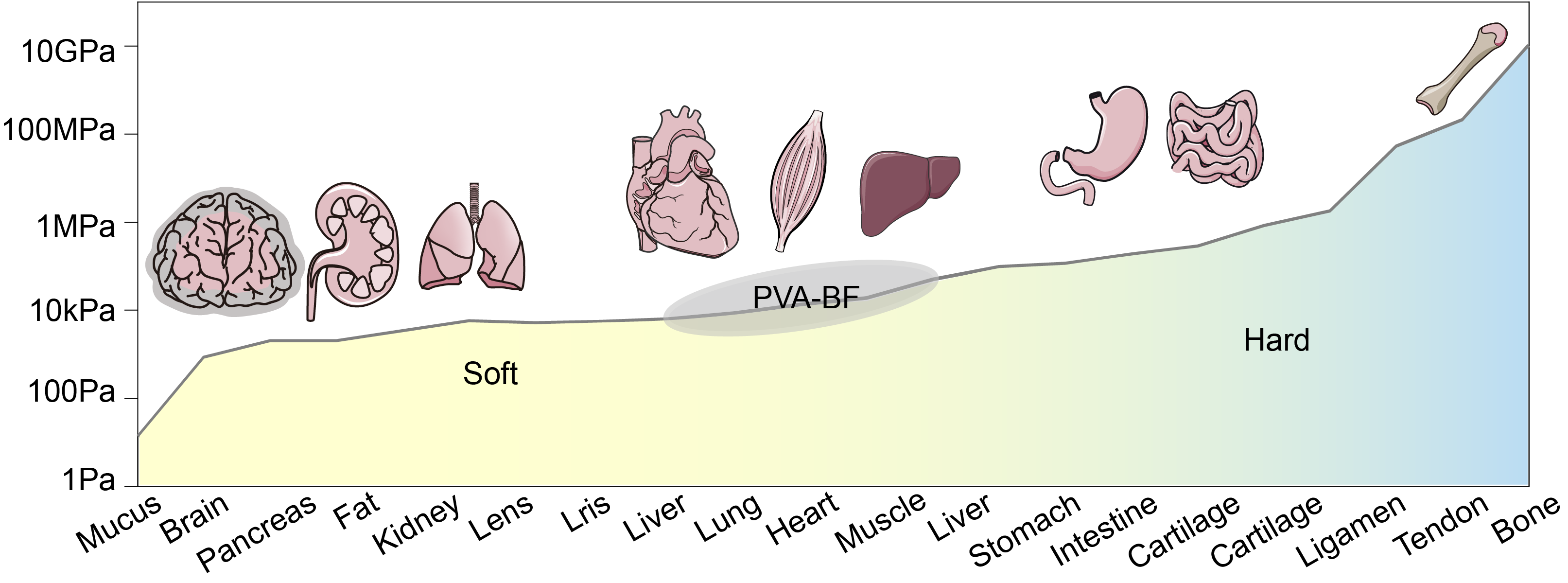
**

**Figure S6. The Young 's modulus of PVA-BF is similar to that of human muscle tissue, which can be applied to muscle sensing for better electromechanical conversion.**


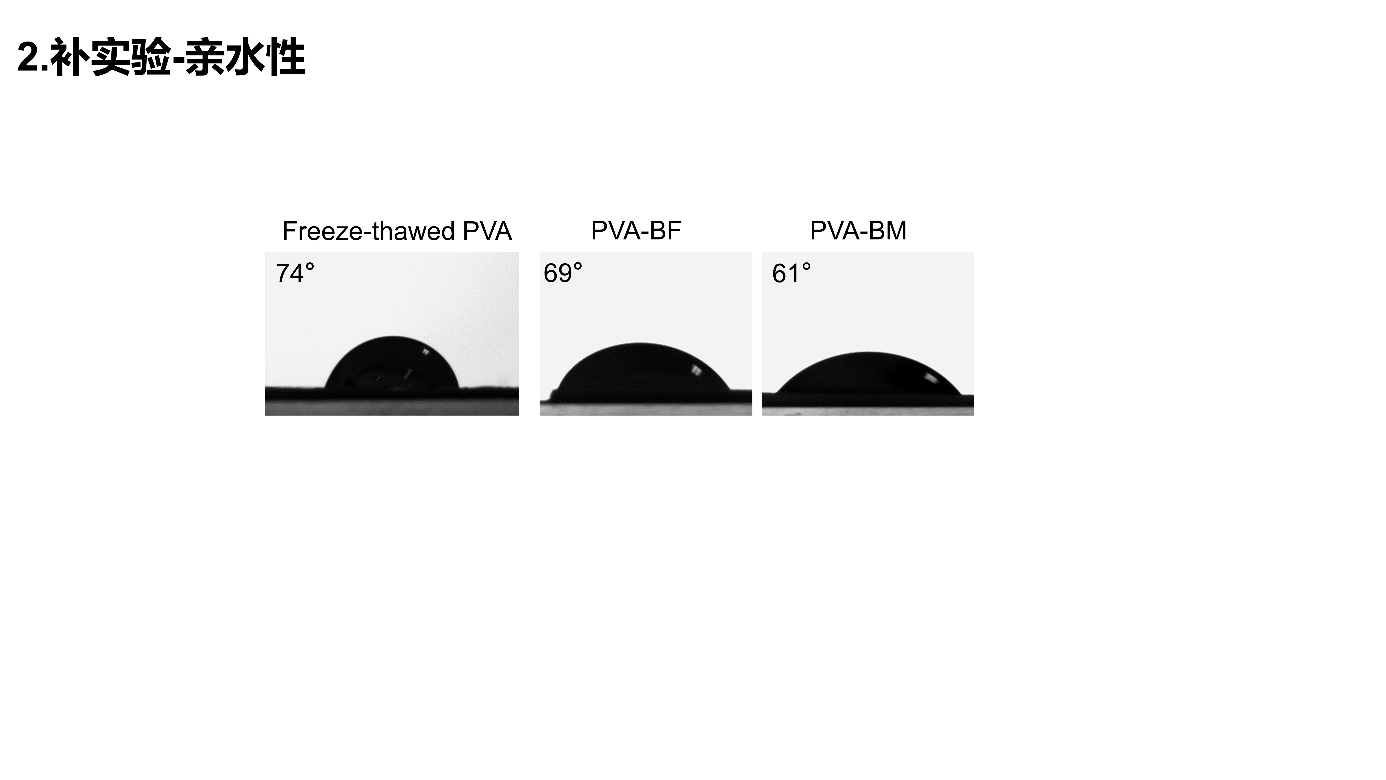


**Figure** **S7. Comparison of water contact angle of freeze-thawed pure PVA, PVA-BF and PVA-BM blends.** From the figure, it can be seen that the contact angle of PVA after protein addition is smaller than that of pure PVA after freezing and thawing, indicating that the hydrophilicity of PVA after protein addition becomes better.


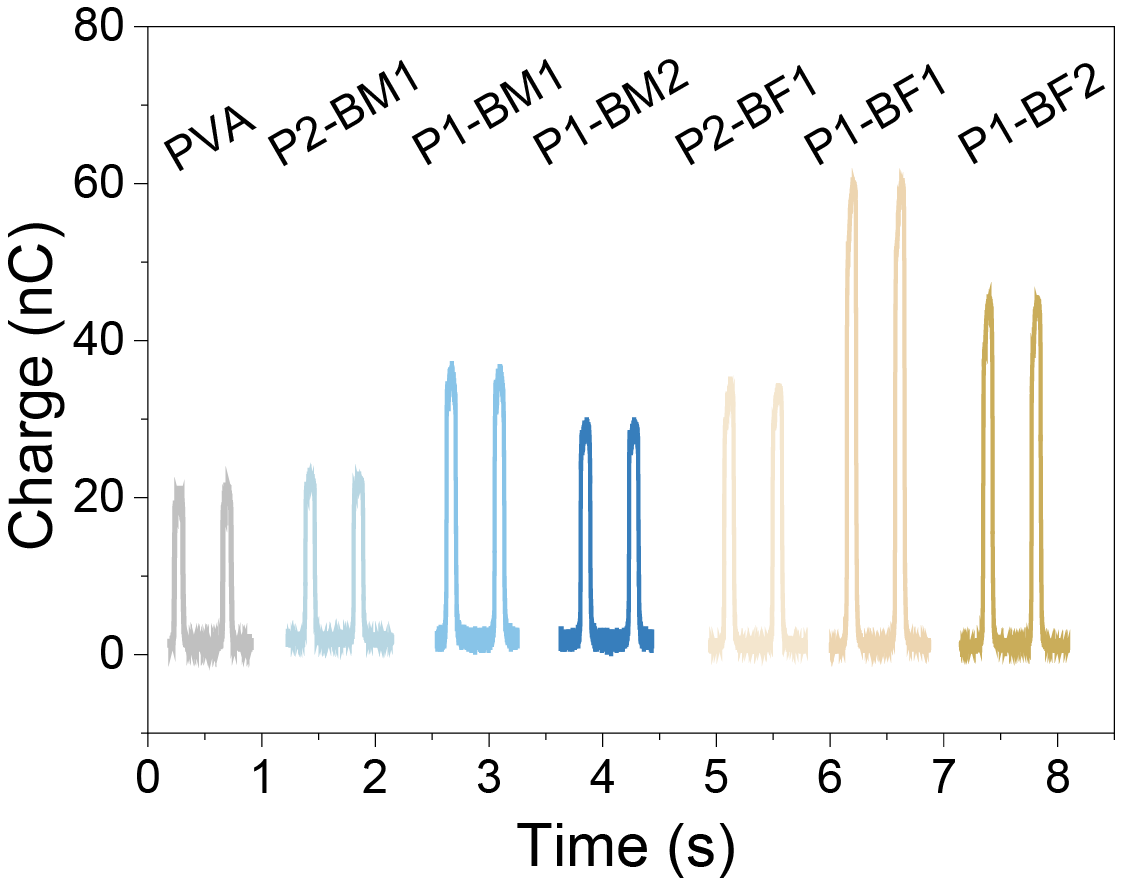


**Figure** **S8. Electrical properties of different films.** Compared to PVA, protein-incorporated PVA transferred more charge, and PVA-BF transferred more charge than PVA-BM, with PVA1-BF1 transferring nearly 3-fold more charge than PVA (21.5 nC to 60.8 nC).





**Figure S9.** **I and Q output of PVA1-BF1 at 0.5-1Hz.**

**
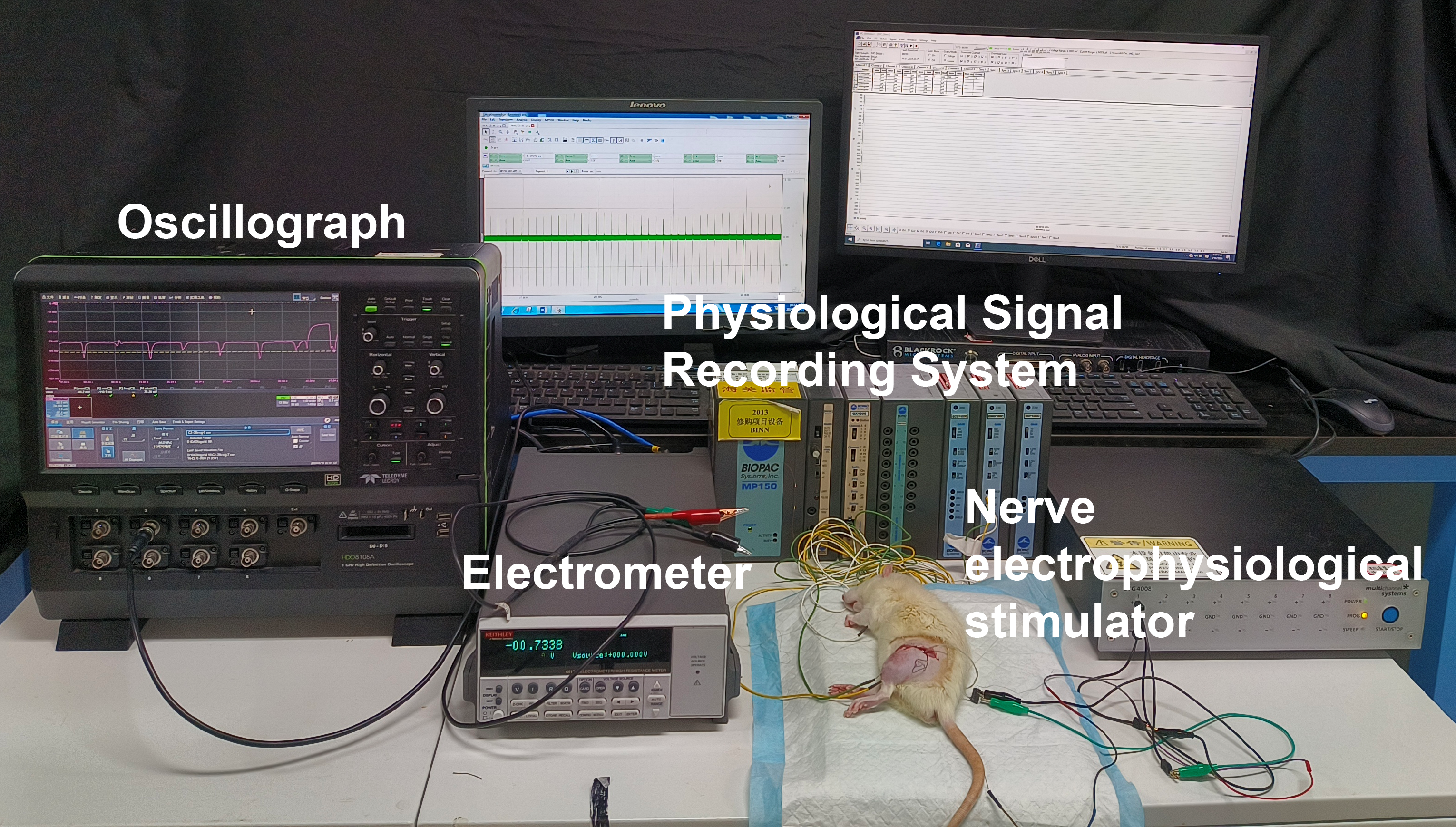
**

**Figure S10. The instrument and connection of muscle force sensing experiment.** External electrical stimulation was provided to the leg nerves of rats by an electrical stimulation instrument, and EMG signals were recorded by MP150 (Biopac, USA). At the same time, the bi-TENG is connected with the electrometer and the corresponding electromechanical conversion signal is displayed by the oscilloscope.

**

**

**Figure S11.** **Cell morphology of L929 cells after culturing on the surface of different materials. a)** Cultured for 1 day. **b)** Cultured for 3 days.

**
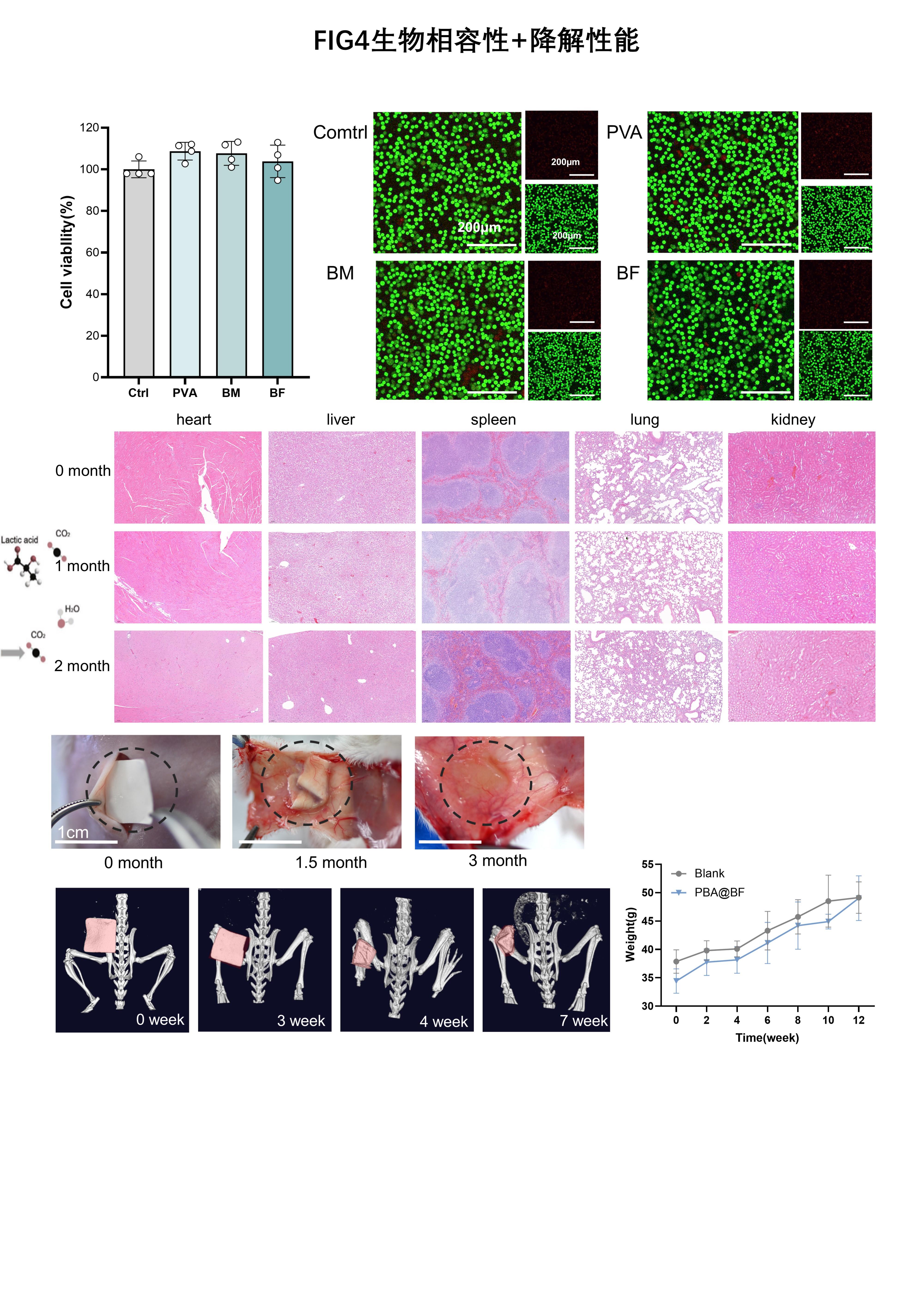
**

**Figure S12. Morphological changes of PVA1-BF1 films (1x1cm) implanted subcutaneously in the back of mice for three months.**


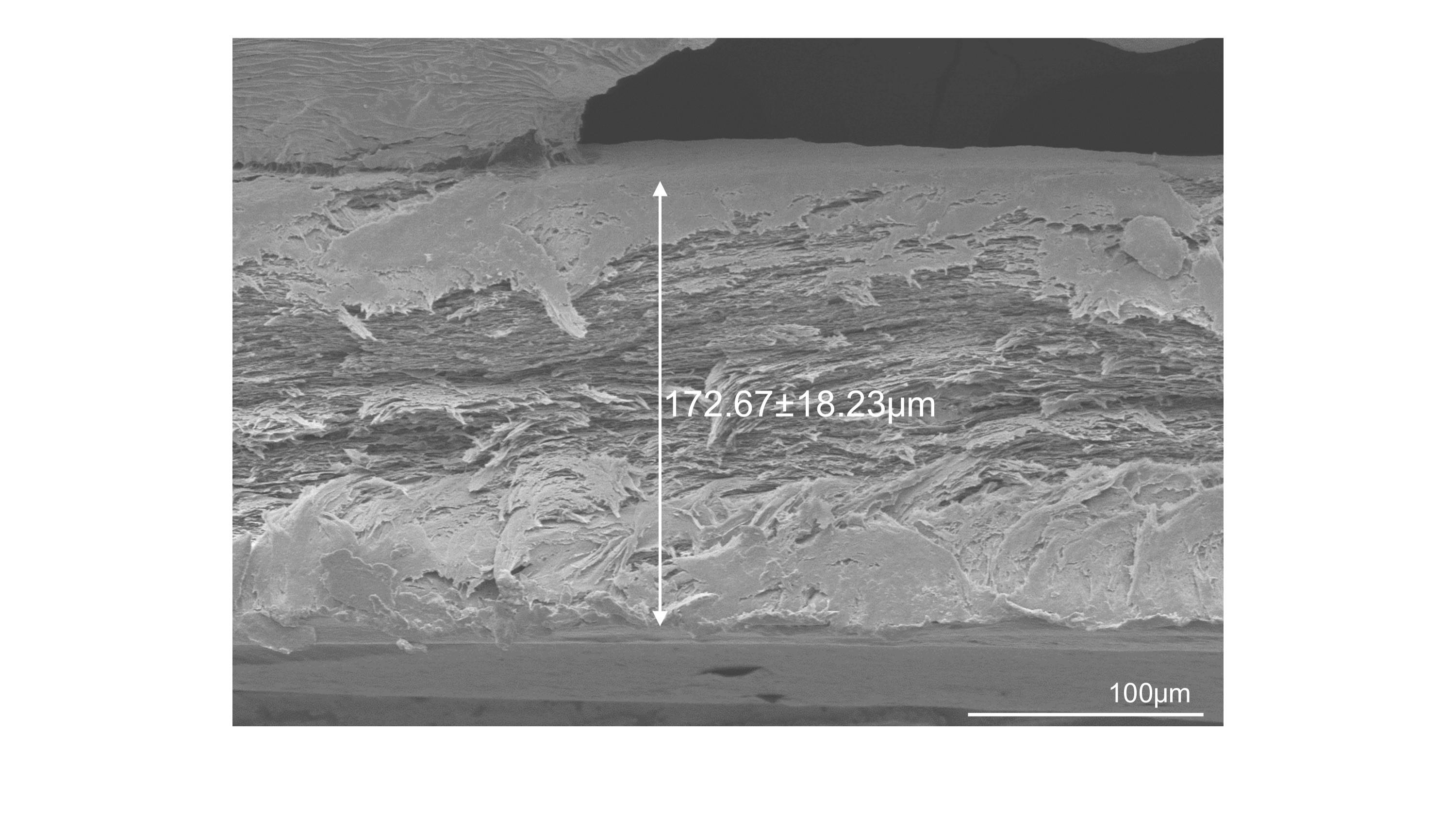


**Figure S13. The thickness of the BF-PVA porous film was measured by SEM in several shots, after calculating its mean and standard deviation as 172.67 ± 18.23 μm .**

**
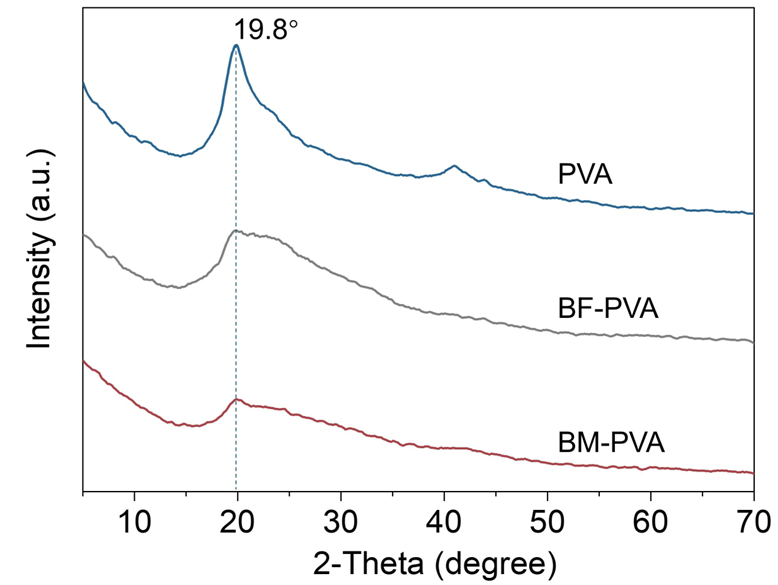
**

**Figure S14. The XRD pattern shows that the peaks BF-PVA and BM-PVA at 19.8° are significantly less intense than PVA, which indicates a decrease in crystallinity.**

**
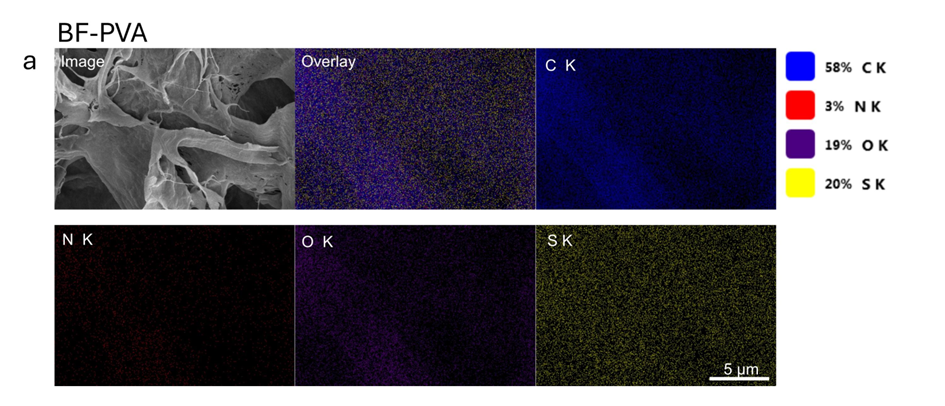
**


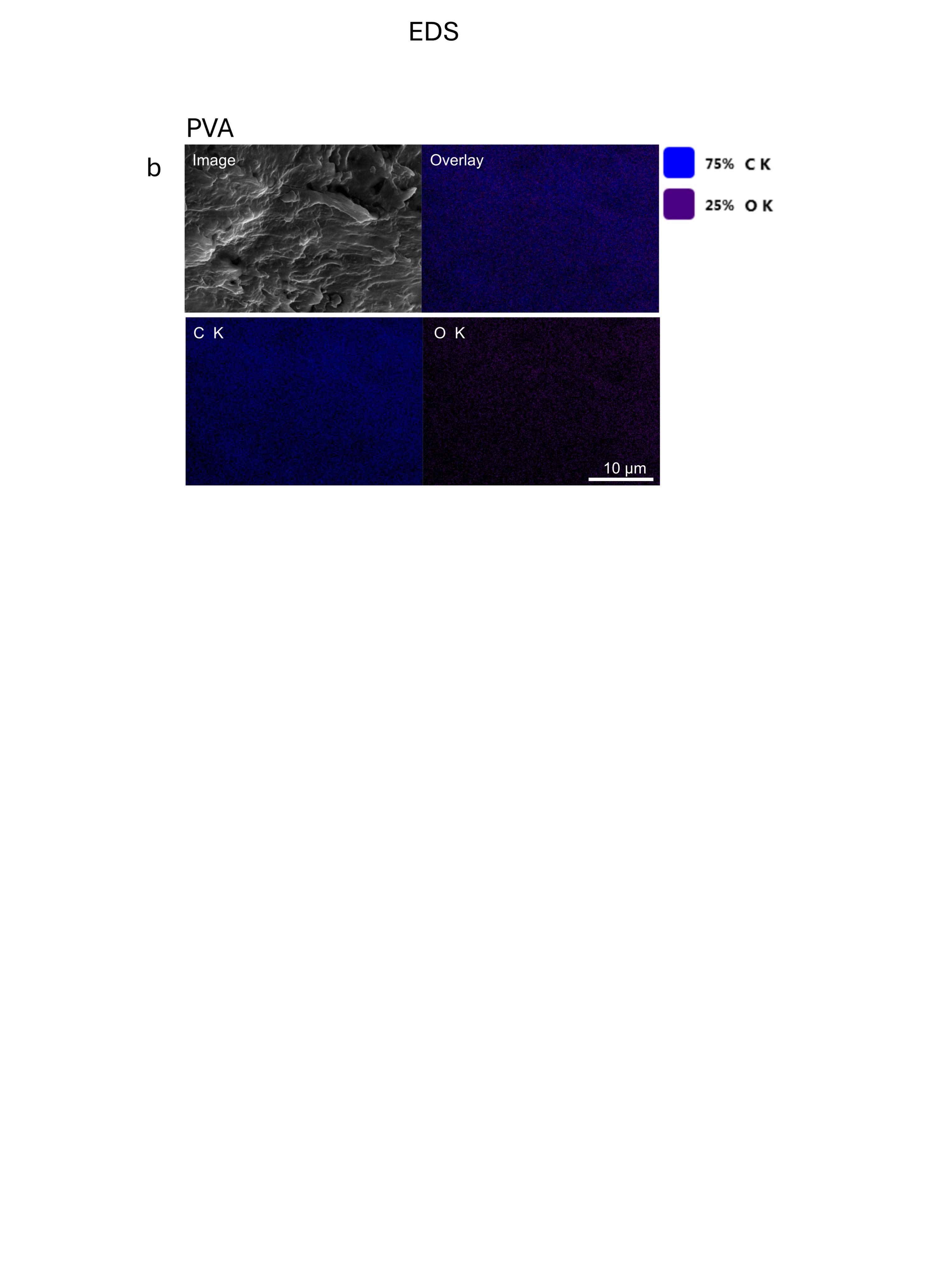


**Figure S15. EDX images of PVA and BF-PVA and the corresponding elemental occupancies. Due to the addition of BF, BF-PVA has more nitrogen (N) and sulfur (S) than PVA.**

**
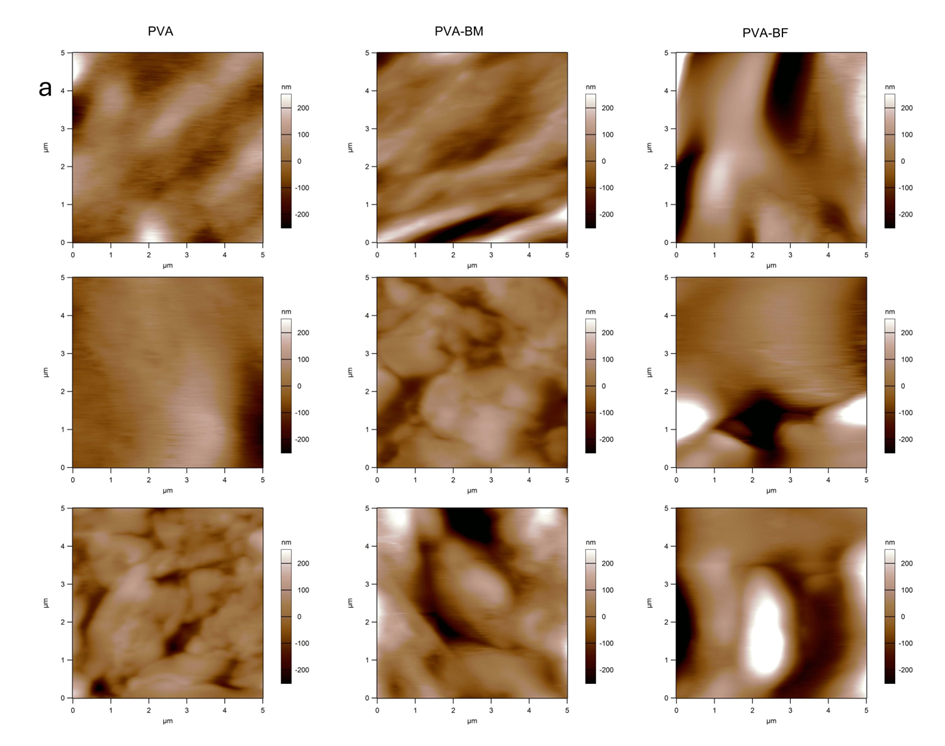
**


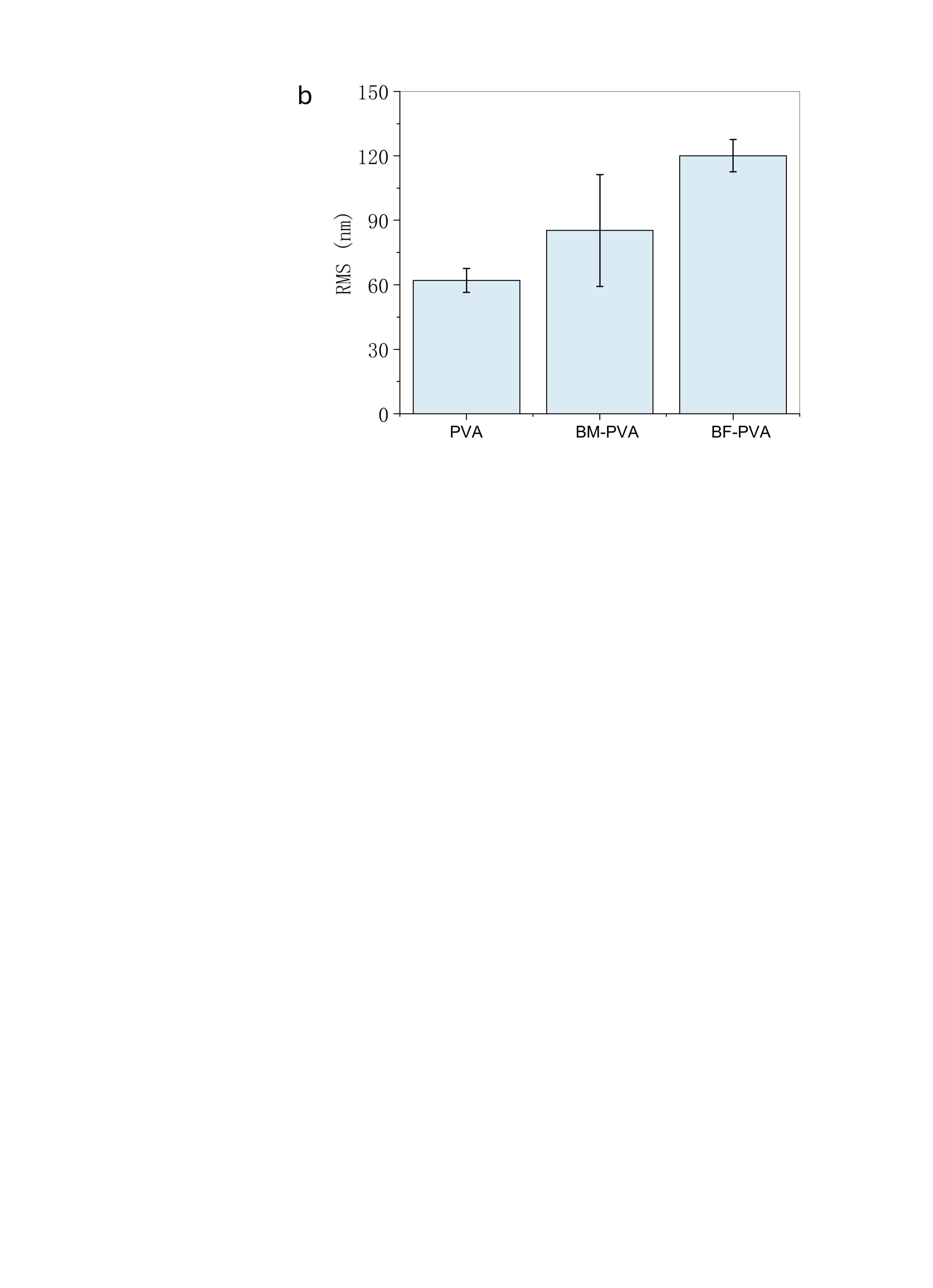


**Figure S16. a)AFM images of PVA、BM-PVA and BF-PVA. b) Average surface roughness of PVA、BM-PVA and BF-PVA.**


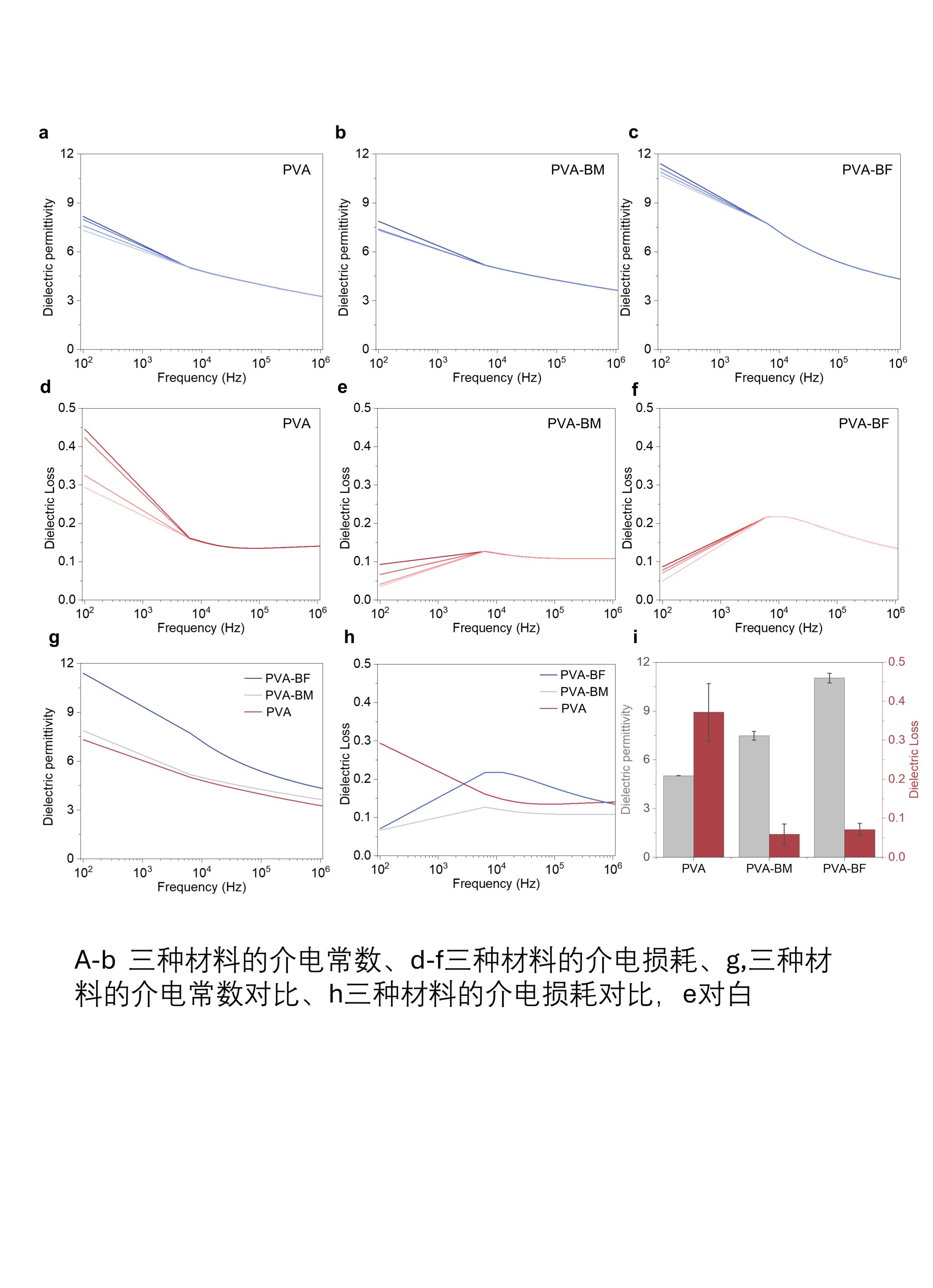


**Figure S17. a-c)** **Dielectric permittivity of PVA, BF-PBA and PVA-BF. d-f) Dielectric loss of PVA, BF-PBA and PVA-BF. g)** **Comparison of dielectric constants of PVA, BF-PBA and PVA-BF. h) Comparison of dielectric loss of PVA, BF-PBA and PVA-BF. i) Statistical comparison at low frequencies (100 Hz)**

**
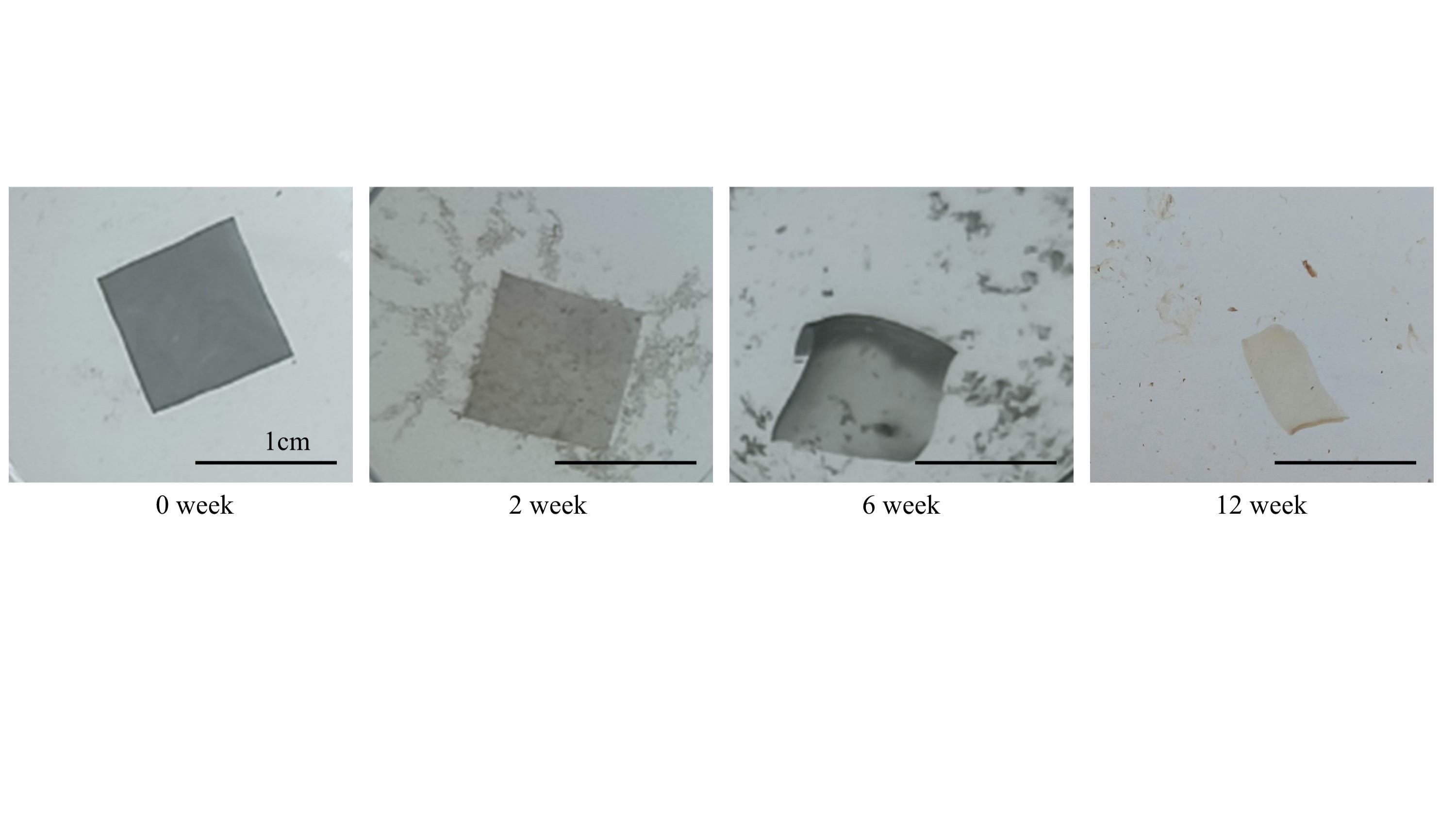
**

**Figure S18. Photograph of the degradation of BF-PVA.** **BF-PVA was immersed in phosphate buffer saline (1xPBS) at a constant temperature of 37°C for 3 months for recording.**

**
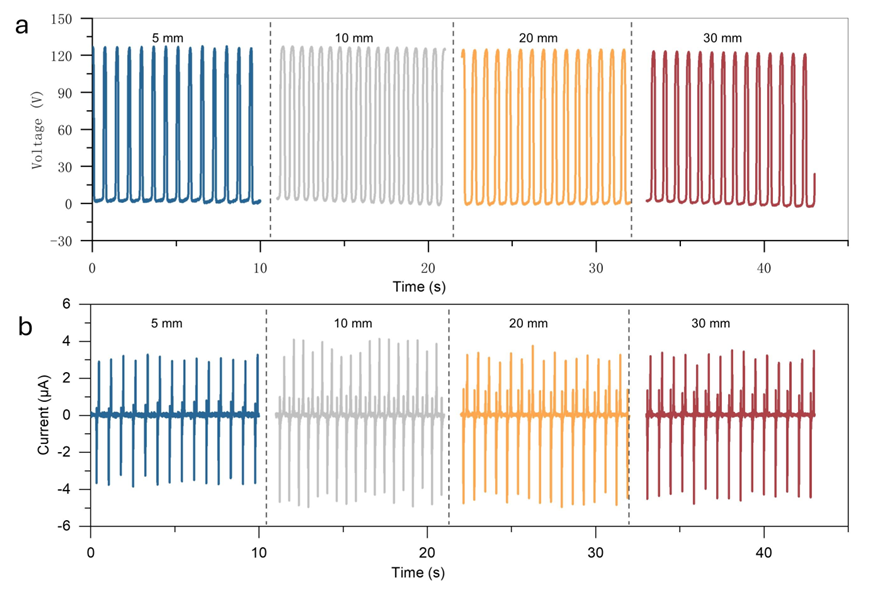
**

**Figure S19.** **Effect of contact-separation distance on output.**

**
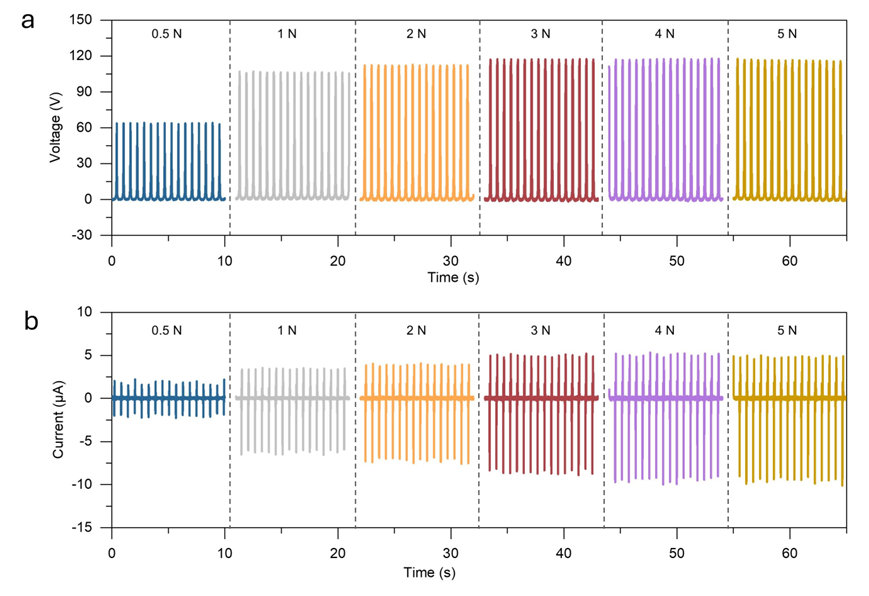
**

**Figure S20.** **Effect of input pressure on output.**

**
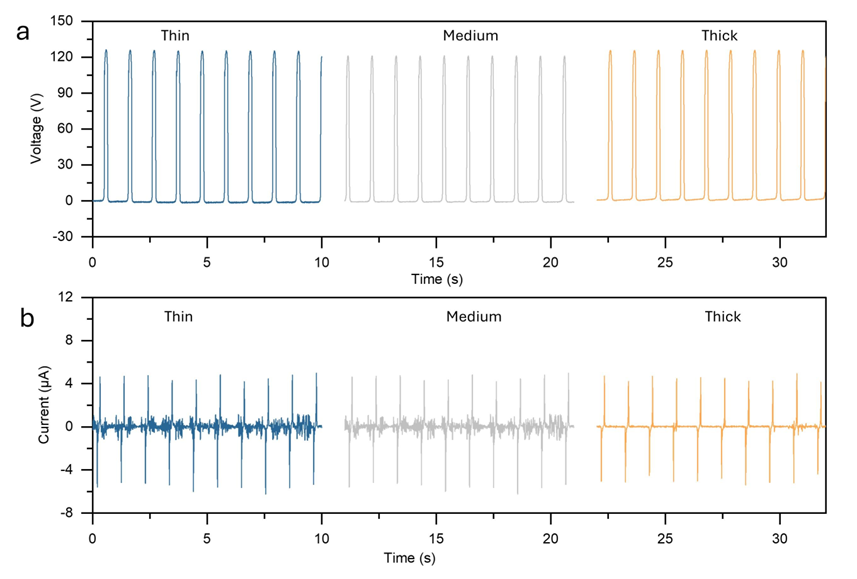
**

**Figure S21.** **Effect of film thickness on output.**

| **Input** | | **Variant** | | **Voltage (V)** | **Current(μA)** |
| --- | --- | --- | --- | --- | --- |
| Distance | 5 mm | | | 126.51±0.76 | 12.27±0.33 |
|  | 10 mm | | | 125.95±0.53 | 14.98±0.96 |
|  | 20 mm | | | 124.03±0.26 | 15.47±0.74 |
|  | 30 mm | | | 122.40±0.42 | 14.78±0.66 |
| Power | | 0.5 N | | 63.65±0.54 | 2.64±0.19 |
|  |  | 1 N | | 106.52±0.46 | 7.85±0.31 |
|  |  | 2 N | | 112.33±0.33 | 9.13±0.36 |
|  |  | 3 N | | 117.06±0.27 | 10.65±0.19 |
|  |  | 4 N | | 117.37±0.40 | 11.92±0.39 |
|  |  | 5 N | | 116.78±0.71 | 12.08±0.28 |
| Film thickness | | | Thin (105.34±3.21 μm) | 125.32±0.27 | 14.24±0.98 |
|  |  |  | Medium (182.67±4.16 μm) | 120.34±0.27 | 13.77±0.76 |
|  |  |  | Thick (498.67±21.36 μm) | 125.32±0.33 | 12.65±0.86 |

**Table S1. Effect of film thickness/input pressure/distance on output**

**Video S1.** In the video, it is first shown that the PVA porous film exhibits brittleness and fracture when bent, and then it is shown that the BF-PVA exhibits good flexibility when bent at the same time, and there is no crack after fully folded.
